# Long-Term Culture of Genome-Stable Bipotent Stem Cells from Adult Human Liver

**DOI:** 10.1016/j.cell.2014.11.050

**Published:** 2015-01-15

**Authors:** Meritxell Huch, Helmuth Gehart, Ruben van Boxtel, Karien Hamer, Francis Blokzijl, Monique M.A. Verstegen, Ewa Ellis, Martien van Wenum, Sabine A. Fuchs, Joep de Ligt, Marc van de Wetering, Nobuo Sasaki, Susanne J. Boers, Hans Kemperman, Jeroen de Jonge, Jan N.M. Ijzermans, Edward E.S. Nieuwenhuis, Ruurdtje Hoekstra, Stephen Strom, Robert R.G. Vries, Luc J.W. van der Laan, Edwin Cuppen, Hans Clevers

**Affiliations:** 1 Hubrecht Institute-KNAW, University Medical Centre Utrecht, CancerGenomics.nl, Uppsalalaan 8, 3584 CT Utrecht, the Netherlands; 2 Department of Surgery, Erasmus MC-University Medical Center, Postbus 2040, 3000 CA Rotterdam, the Netherlands; 3 Surgical Laboratory, Tytgat Institute for Liver and Intestinal Research, Academic Medical Center, Meibergdreef 9, 1105 AZ Amsterdam, the Netherlands; 4 Division of Pediatric Gastroenterology, Wilhelmina Children’s Hospital, University Medical Center Utrecht, Lundlaan 6, 3584 EA Utrecht, the Netherlands; 5 Department of Clinical Chemistry and Haematology, University Medical Center Utrecht, Lundlaan 6, 3584 EA Utrecht, the Netherlands; 6 Division of Pathology, Department of Laboratory Medicine, Karolinska Institute, Alfred Nobels Alle 8, F 56 141-86 Stockholm, Sweden; 7 Unit for Transplantation Surgery, Department of CLINTEC, Karolinska Institute, Karolinska University Hospital Huddinge, Hälsovägen, Flemingsberg, SE-141 86 Stockholm, Sweden; 8 Hubrecht Organoid Technology (HUB), Uppsalalaan 8, 3584CT, Utrecht, the Netherlands

## Abstract

Despite the enormous replication potential of the human liver, there are currently no culture systems available that sustain hepatocyte replication and/or function in vitro. We have shown previously that single mouse Lgr5+ liver stem cells can be expanded as epithelial organoids in vitro and can be differentiated into functional hepatocytes in vitro and in vivo. We now describe conditions allowing long-term expansion of adult bile duct-derived bipotent progenitor cells from human liver. The expanded cells are highly stable at the chromosome and structural level, while single base changes occur at very low rates. The cells can readily be converted into functional hepatocytes in vitro and upon transplantation in vivo. Organoids from α1-antitrypsin deficiency and Alagille syndrome patients mirror the in vivo pathology. Clonal long-term expansion of primary adult liver stem cells opens up experimental avenues for disease modeling, toxicology studies, regenerative medicine, and gene therapy.

## Highlights

• Establishment of a long-term human liver organoid culture
• Human liver stem cells retain genetic stability after long-term expansion
• Liver organoid cultures differentiate to functional hepatocytes in vitro and in vivo
• Organoids derived from patients with genetic disorders model liver disease in vitro

## Graphical Abstract

**Figure:**
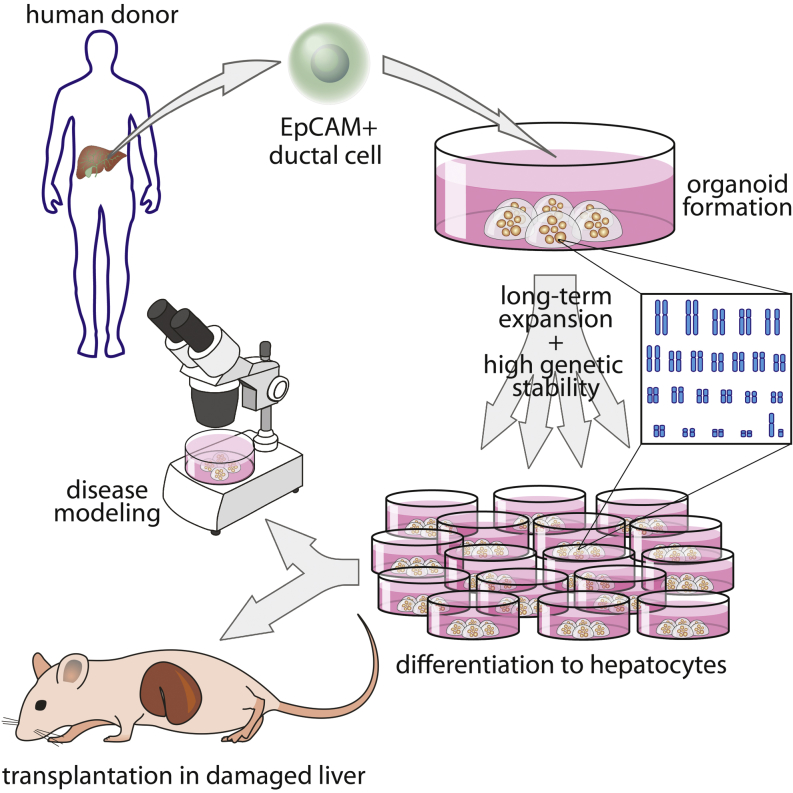

## Teaser

A culture system that allows long-term expansion of human liver can be used to model and study various human diseases.

## Introduction

The liver is mainly composed of two epithelial cell types, hepatocytes and ductal cells. Hepatocytes synthesize essential serum proteins, control metabolism, and detoxify a wide variety of endogenous and exogenous molecules (Duncan et al., 2009). Despite their considerable replication capacity in vivo (Michalopoulos, 2014), hepatocytes have resisted long-term expansion in culture (Mitaka, 1998). Indeed, a recent study describes a human liver hepatocyte culture system for a period of ∼1 week with only 10-fold expansion (Shan et al., 2013). As an alternative, human embryonic stem (hES) cells and human induced pluripotent stem (hiPS) cells have been differentiated toward hepatocyte-like cells. However, recent reports imply that genetic and epigenetic aberrations occur during the derivation and reprogramming processes (Liang and Zhang, 2013; Pera, 2011; Lund et al., 2012). These range from chromosomal abnormalities (Laurent et al., 2011),“de novo” copy number variations (CNVs) (Hussein et al., 2011), and point mutations in protein-coding regions (Gore et al., 2011). Such changes may complicate their use for regenerative medicine purposes (Bayart and Cohen-Haguenauer, 2013).

We have recently described a culture system that allows the long-term expansion (>1 year) of single mouse adult intestine (Sato et al., 2009), stomach (Barker et al., 2010), liver (Huch et al., 2013b), and pancreas (Huch et al., 2013a) stem cells. *Lgr5*, the receptor for the Wnt agonists R-spondins (Carmon et al., 2011; de Lau et al., 2011), marks adult stem cells in these mouse tissues (Barker et al., 2007, 2010; Huch et al., 2013a, 2013b). These cultures remain committed to their tissue of origin. We have recently adapted the technology to allow culturing of human intestinal stem cells (Jung et al., 2011; Sato et al., 2011) and have shown that patient-derived intestinal organoids recapitulate the pathology of hereditary intestinal diseases (Bigorgne et al., 2014; Dekkers et al., 2013; Wiegerinck et al., 2014). Here, we pursue the establishment of an organoid culture system for human liver.

## Results

### Optimization of Human Liver Stem Cell Culture

**Figure 1:**
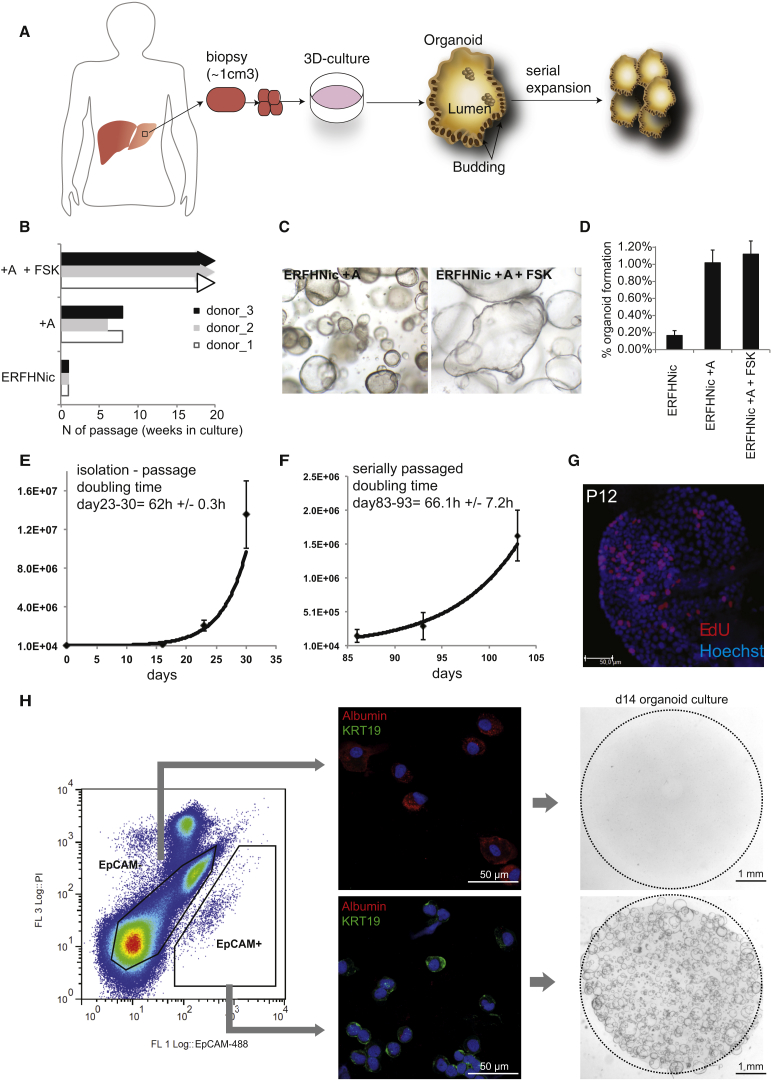
Growing Liver Organoids from Ductal Cells 3,000 or 10,000 human primary liver cells were seeded per well in a 48-well plate in different culture conditions, as indicated. (A) Scheme of the experimental protocol. (B) Mouse liver culture medium (ERFHNic) or medium supplemented with A8301 (A) or A8301 and Forskolin (FSK). The cultures were split every week 7–10 days at a ratio of 1:4−1:6 dilution. Supplementing with A8301 and FSK significantly increased the expansion efficiency to grow for >18 passages at a split ratio of 1:4–1:6 every 7–10 days for >5 months. Experiments were performed in triplicate. Each bar indicates a different donor. (C) DIC images of organoids treated with mouse liver medium with A8301 and with (right) or without (left) FSK. Magnification, 4×. (D) Percentage of colony formation efficiency in the presence or absence of A8301 and/or FSK. Experiments were performed in triplicate and for five donors. Results are expressed as mean ±SEM of five independent experiments. (E–G) Expansion rates, in vitro growth curves, and EdU incorporation were analyzed at early and late passages in EM. (E and F) Graphs illustrate the number of cells counted per well at each passage from P1–P4 (E) to P16–P18 (F). Results are expressed as mean ±SEM of three independent cultures. The doubling time was calculated as described in the Extended Experimental Procedures. (G) EdU incorporation was still detected at late passages. (H) Human liver cell suspensions were separated into EpCAM+ ductal cells and larger EpCAM− hepatocytes (for exact gating strategy, see Figure S2C). Identity of the populations was confirmed by staining for Albumin and KRT19. Sorted cells were grown for 14 days. Organoids were exclusively derived from EpCAM+ ductal cells. See also Figures S1 and S2.

**Figure S1:**
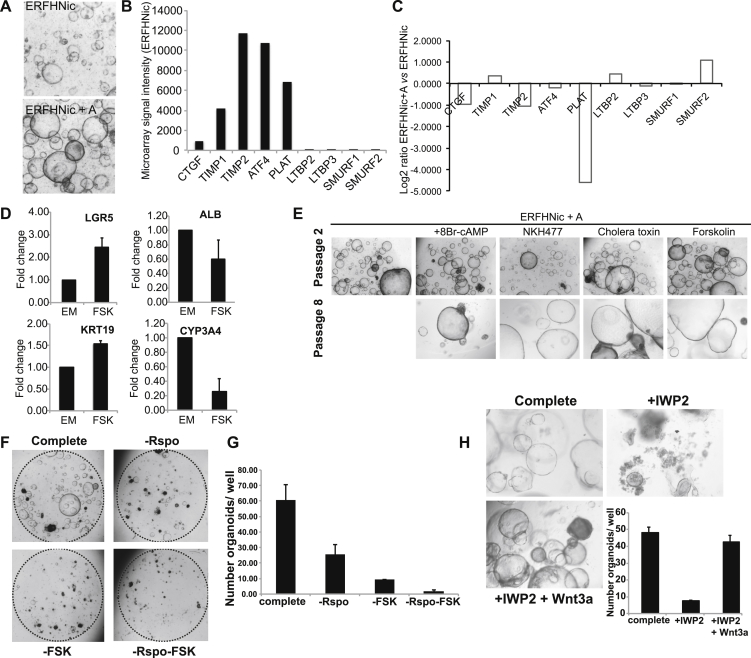
TgFb Inhibition, Active Wnt Signaling, and cAMP Activation Are Essential for the Long-Term Expansion of Human Liver Cells, Related to Figure 1 (A–E) Liver tissue was digested using collagenase dissociation as described in Material and Methods. Single cell suspensions were counted and 3000 or 10000 cells were seeded per well in a 48well plate. Cells were cultured in mouse liver medium containing Egf, Rspo, Fgf10, Hgf and Nicotinamide (ERFHNic) or the same medium supplemented with A8301 (+A) or forskolin (+FSK) or the indicated compounds. (A) Representative images of organoid cultures grown in the mouse medium (ERFHNic) or medium supplemented wiht A8301 (+A). (B) Gene expression of TGFb target genes, sequesters and inhibitors in 2 weeks old cultures maintained in mouse medium. Results are expressed as microarray signal of the specific gene after normalization. (C) Gene expression of the specific TGFb genes downregulated upon A8301 treatment. Results are expressed as log2 fold change when comparing cutlures treated vs non-treated. (D) Gene expression of LGR5, KRT19, ALB and CYP3A4 upon FSK treatment. (E) Images of organoid cultures treated for up to 8 passages with the indicated cAMP activators. (F) Expanding human liver organoids grown in complete medium as described in Methods were maintained in that medium (complete) or transferred to a medium without Rspo (-Rspo), without FSK (-FSK) or both (-Rspo-FSK). After withdrawal the cultures deteriorated and could no longer be passaged. Representative image of 1 donor material 7 days after withdrawal. (G) Quantification of the number of organoids per well after a 7 days withdrawal of Rspo or FSK or both. Results are expressed as the mean +/- SEM of 2 independent human donor material and 2 independent experiements. (H) Addition of the porcupine inhibitor (IWP2) to the medium resulted in growth arrest evident as early as 5 days after the treatment. That effect could be rescued by the exogenous addition of Wnt into the medium (+Wnt3a). Representative images taken 12 days after treatment. Organoid numbers were counted 12 days after the treatment. Graph indicating the number of organoids in the presence/absence of the indicated compounds. Results are expressed as mean +/- SEM of 2 independent human liver cultures.

Our defined mouse liver medium (ERFHNic [Huch et al., 2013b]) supported the growth of human liver cells only for 2–3 weeks (Figure 1A and 1B and Figure S1A, top, available online). Gene expression profiles of human liver cultures that were maintained for 2 weeks in “mouse liver medium” revealed highly active Tgf-β signaling. Tgf-β target genes such as *CTGF*, *PLAT*, *TIMP1*, and *TIMP2* were highly expressed, whereas Tgf-β sequesters (*LTBP2* and *LTBP3*) and Smad4 inhibitors (*SMURF1* and *SMURF2*) (Massagué et al., 2005) were virtually absent (Figure S1B). Tgf-β signaling induces growth arrest and epithelial-to-mesenchymal transition (Xu et al., 2009). Specific inhibition of Tgf-β receptors Alk4/5/7 by the small molecule inhibitor A8301 downregulated *CTGF*, *TIMP2*, and *PLAT* (Figure S1C), extended the time in culture (∼6–7 weeks, six to seven splits) (Figure 1B), and enhanced colony-forming efficiency (Figure 1D). Still, the cultures eventually deteriorated (Figures 1B and 1C, left). Expression of the stem cell marker *LGR5* decreased over time, whereas differentiation markers such as Albumin (*ALB*) or *CYP3A4* were upregulated (data not shown), indicating that our conditions were promoting differentiation.

We then tested additional compounds to induce proliferation and/or *LGR5* expression (Table S1). Proliferating bile-duct progenitor cells occur both during homeostasis (Furuyama et al., 2011) and after damage (Dorrell et al., 2011; Huch et al., 2013b; Shin et al., 2011). As Forskolin (FSK), a cAMP pathway agonist, induces proliferation of biliary duct cells in vivo (Francis et al., 2004), we asked whether cAMP would support the human liver cultures.

FSK addition upregulated *LGR5* and the ductal marker *KRT19*, whereas *ALB* and *CYP3A4* decreased (Figure S1D). Colony-forming efficiency was essentially unchanged (Figure 1D), yet the cultures expanded as budding organoids for many months in culture (>6 months) at a weekly split ratio of 1:4–1:6 (Figures 1B and 1C, right). Similar results were observed with other cAMP agonists (8-BrcAMP, Cholera toxin or NKH477) (Figure S1E). Removal of cAMP agonists resulted in rapid deterioration (Figures S1F and S1G). Similarly, removal of the Wnt agonist R-spo or blocking Wnt secretion by porcupine inhibition (IWP-2) resulted in rapid loss of the cultures (Figures S1F–S1H). This effect was rescued by exogenous addition of Wnt (Figure S1H). Twelve additional healthy human donor liver biopsies were cultured in the improved medium, with a consistent doubling time of ∼60 hr, independent of the age of the culture (Figures 1E and 1F and Table S2). EdU incorporation confirmed that the cells maintained their proliferative state in vitro (Figure 1G) for >3 months. Cultures could be readily frozen and thawed (data not shown). Thus, Wnt signals, cAMP activation, and Tgf-β inhibition were essential for long-term expansion.

### Organoids Originate from Ductal cells

**Figure S2:**
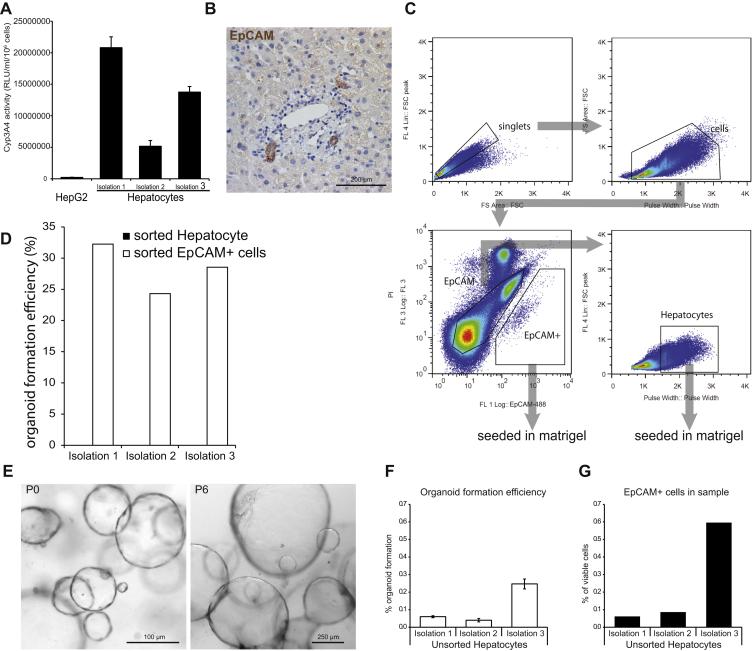
Human Liver Cultures Are of Ductal Origin, Related to Figure 1 (A) Cyp3A4 activity of Percoll purified primary human hepatocytes after 4 days in culture in comparison to HepG2 cells (Mean ± SEM of 3 replicates). (B) EpCAM marks bileducts in human liver sections. Hepatocytes are EpCAM negative. (C) sorting strategy to purify EpCAM+ ductal cells and Hepatocytes. In the first step, singlets were gated to avoid contamination by cell aggregates. Subsequently, large debris and erythrocytes were excluded. From this population, EpCAM+ PI- (viable) cells were sorted as the ductal population. For hepatocyte sorting large EpCAM- cells were selected. (D) Organoid formation efficiency of sorted ductal and hepatocyte populations after 14 days. Organoids bigger than 100 μm were scored. (E) EpCAM+ sort derived organoids at passage 0 and passage 6. (F) Organoid formation efficiency of unsorted, Percoll purified hepatocytes (Mean ± SEM of 3 replicates) and (G) the respective percentage of residual EpCAM+ cells.

Collagenase perfusion of donor livers yields high numbers of fresh, viable, and functional human hepatocytes (Gramignoli et al., 2012) (Figure S2A). We employed EpCAM to differentially sort hepatocytes (EpCAM^−^) from ductal EpCAM^+^ ductal cells (Figures 1H, S2B, and S2C) (Schmelzer et al., 2007; Yoon et al., 2011). Although hepatocytes formed no organoids, EpCAM^+^ bile duct cells developed into organoids with a striking efficiency of 28.4% ± 3.2% (Figures 1H, S2D, and S2E). Crude liver cell preparations grew into organoid structures with an efficiency that equaled the number of EpCAM^+^ cells (Figures S2F and S2G). In our culture system, ductal cells rather than hepatocytes initiate organoids.

### Clonal Organoids Are Genetically Stable

**Figure 2:**
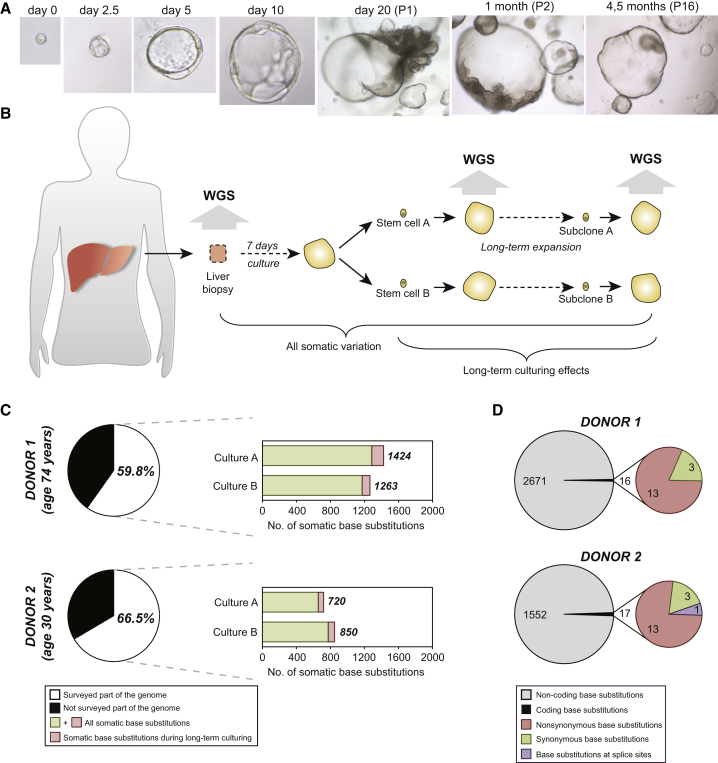
Human Organoids Are Genetically Stable in Culture (A) Clonal cultures were obtained by seeding sorted cells at one cell per well. DIC images at magnifications: 40× (days 0–10), 4× (day 20 onward). (B) Schematic overview of the experimental setup. Two independent donor liver biopsies were cultured for 1 week. Single cells were then clonally expanded to obtain two independent organoid cultures per donor (cultures A and B). After long-term expansion, a second clonal expansion step was performed. The resulting organoid cultures were subjected to WGS analysis. To obtain all somatic variation, variants were filtered for presence in the original biopsy. To determine the effect of long-term culturing on genomic stability, somatic variation was filtered for presence in earlier passages. (C) The pie chart indicates the percentage of the genome that was surveyed per donor. The right panels indicate the absolute numbers of base substitution observed in the surveyed part of the genome. Indicated are the total number of somatic base substitutions per culture and the number induced by long-term culturing. (D) Left panels indicate the total number of somatic base substitutions per donor, and the right panel indicates those affecting protein-coding DNA. See also Figure S3.

**Figure 3:**
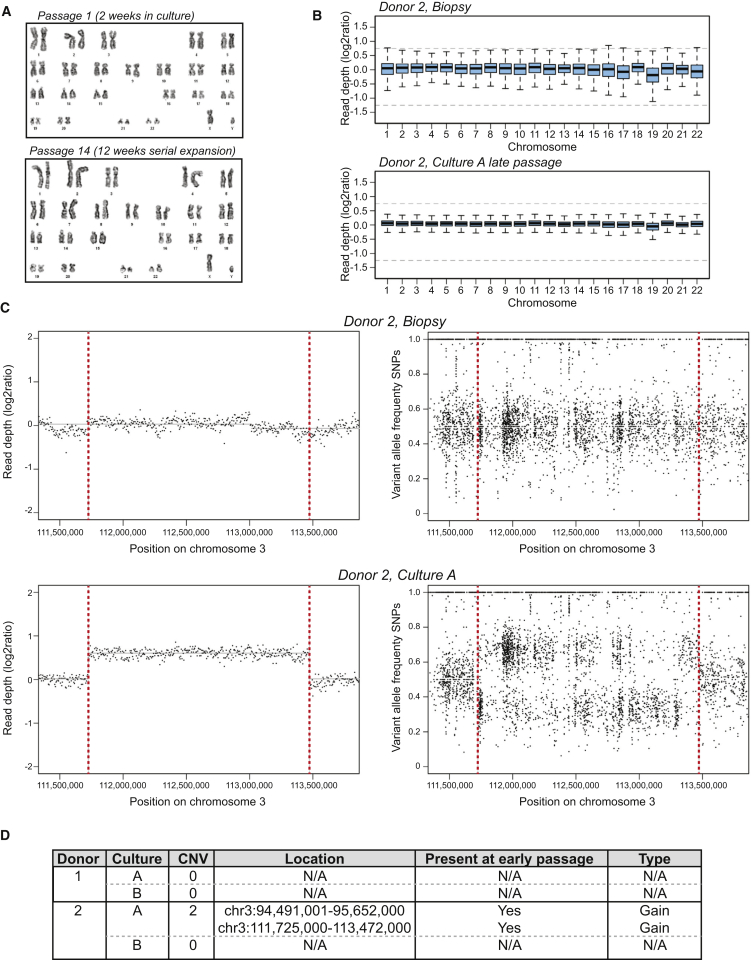
Structural Variation in Human Liver Organoids (A) Representative karyotyping image of organoids cultured for 16 days (P1) and 90 days (P14), illustrating a normal chromosomal count (n = 46). No major chromosomal aberrations were observed in any of the samples analyzed (n = 15). Detailed chromosomal counts for different donors are shown in Figure S4. (B) Read-depth analysis of whole-genome sequencing data over the different chromosomes for the biopsy (top) and organoid culture A (bottom) that were derived from donor 2. Read depth was corrected for GC content and normalized for genome coverage. Gray dotted lines indicate log2 values associated with a gain or deletion. (C) Copy number analysis of a region at chromosome 3 found to harbor a heterozygous gain in culture A of donor 2. Left panels indicate read-depth analysis of the indicated region in 5 kb bins, corrected for GC content and normalized for genome coverage, of the biopsy (top) and organoid culture (bottom). Right panels show the variant allele frequencies of informative nonreference single-nucleotide polymorphisms (SNPs) in the indicated region for the biopsy (top) and organoid culture (bottom). (D) Summary of copy number analysis of the different organoid cultures of the two donors. Somatic CNVs were exclusively observed in culture A derived from donor 2 and were already present in the parental culture. See also Figure S4.

Organoids cultured for 3 months maintained normal chromosome numbers (Figures 3A and S4A). From two donors, we obtained biopsy samples, which we dissociated and cultured in bulk for 7 days. Subsequently, we isolated single cells and established two independent clonal lines for each of the two livers (cultures A and B). After 3 months of expanding these cultures, a second cloning step was performed. We could thus determine all genomic variation accumulated in a single cell during life, derivation, and 3 months of culture (Figures 2A and 2B).

**Figure S3:**
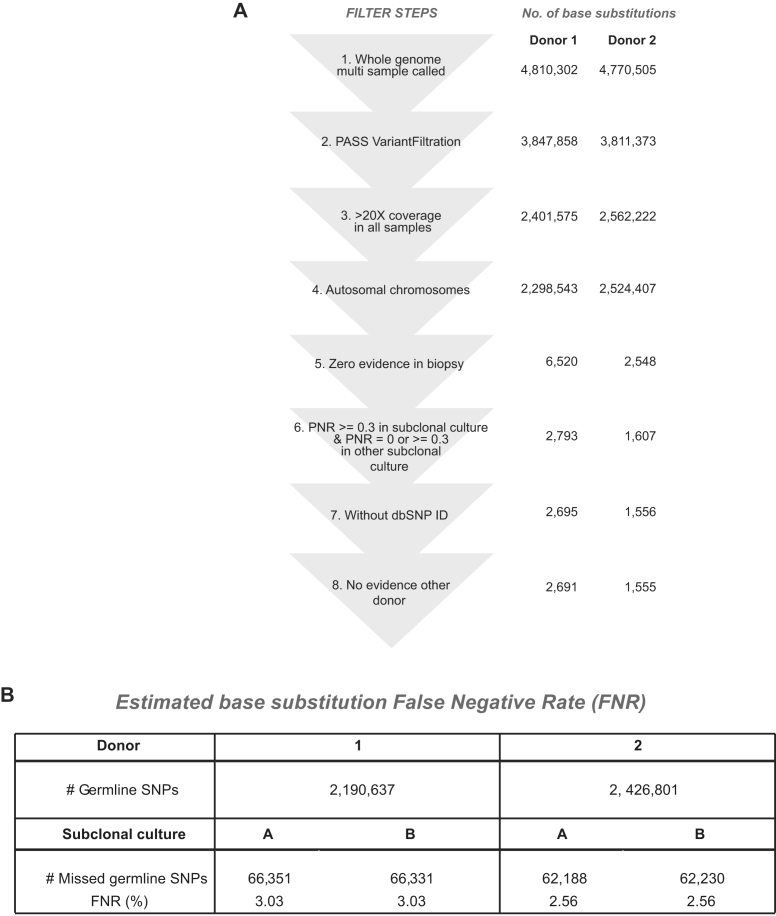
Filtering Steps and FNR of All Sequenced Samples, Related to Figure 2 (A) Total number of base substitutions after various filtering steps: (1) Multi-sample called (biopsy, 2 parental cultures, 2 subclonal cultures for both donors) base substituions with UnifiedGenotyper from GenomeAnalysis toolkit version 2.8-1. (2) First quality control was performed with VariantFiltration from GenomeAnalysis toolkit version 2.8-1 with settings: --clusterWindowSize 10 --filterExpression “MQ0 >= 4 && ((MQ0 / (1.0 ^∗^ DP)) > 0.1)” --filterName “HARD_TO_VALIDATE” --filterExpression “QUAL < 100.0 ” --filterName “LowQual” --filterExpression “QD < 1.5 ” --filterName “LowQD” (3) Base substitutions with a coverage of at least 20X in all samples. (4) Base substitutions at autosomal chromosomes. (5) Base substitutions without any evidence in the biopsy sample (somatic events). (6) Base substitutions were called if PNR >= 0.3 in the subclones. Base substitutions that were called and also have evidence in the other subclone of the same individual are removed if the 0 < PNR < 0.3 and the number of alternative reads is >1. (7) Base substitutions without a dbSNP_137 identifier. (8) Base substitutions without evidence in the other individual. (B) Estimation of False Negative Rate (FNR). The germline SNP sets consist of base substitutions that pass filter steps 1-4, are called in the biopsy of the donor with a PNR >= 0.3 and number of alternative alleles > 1. Subsequently, base substitutions were called in the subclonal cultures that passed filter steps 1-4 and 6. Germline SNPs that were missed in the subclonal cultures are used to calculate the FNR.

We observed 720–1,424 base substitutions per cultures, of which 63–139 were introduced during the 3 months culture (Figure 2C). Therefore, the majority of the base substitutions identified had been incorporated in vivo (during life) or introduced during organoid derivation, but not during culture. How do these numbers compare to published data? iPS cells contain 1,058–1,808 de novo base substitutions (determined at passage numbers between 15 and 25) compared to their parental somatic cells (Cheng et al., 2012). Of note, the numbers from these studies do not include the variation acquired in vivo in the parental somatic cells. Thus, 3 months of in vitro expansion of liver organoids introduces 10-fold fewer base substitutions than iPS cell reprogramming. Of the total number of base substitutions, only few were located in protein-coding DNA (seven to nine base substitutions per culture; Figures 2D and S3). With the exception of one synonymous mutation in culture A from donor 2 (Table S3), all mutations were already present in the early passage clonal cultures, indicating that they were not incorporated during the 3 months of expansion. None of the mutated genes occurs in COSMIC databases (Table S3). In iPS cells, an average of six base substitutions per line affect protein-coding DNA (Cheng et al., 2012; Gore et al., 2011).

**Figure S4:**
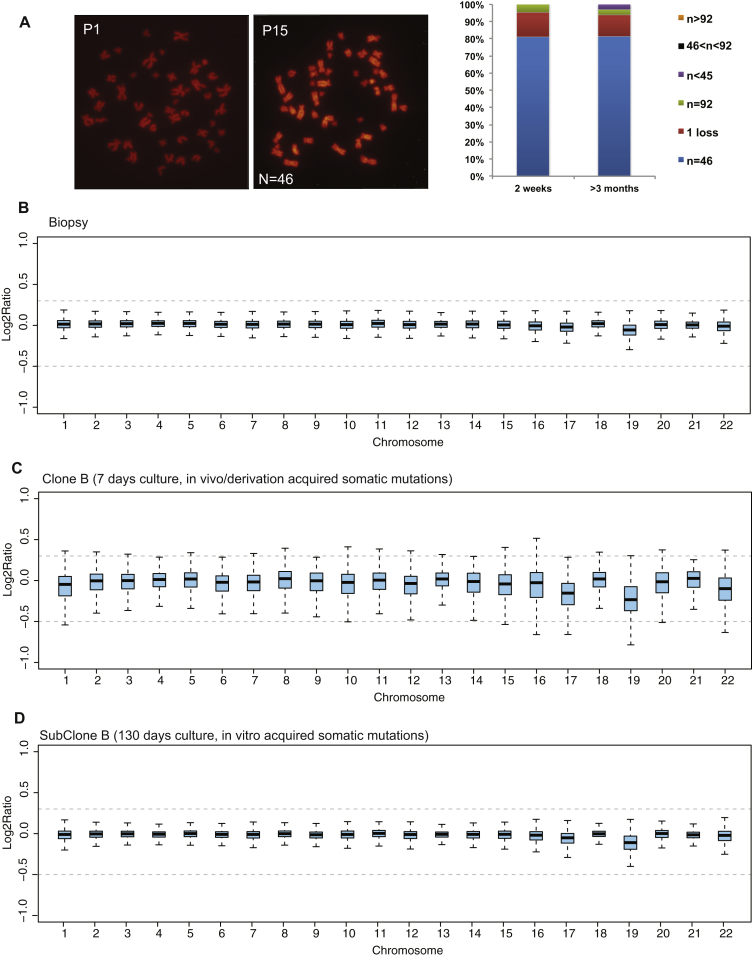
Genetic Stability of Human Liver Stem Cell Cultures, Related to Figure 3 (A) Chromosome numbers were counted on a total of 76 metaphases from 2 different donors. B-D: Genetic stability was evaluated on clonally expanded cultures from 1 donor by WGS. Absence of DNA-Copy number alterations in human liver stem cell cultures clonally expanded long-term in culture. Box plots display the Log2 intensity ratios for the original biospy (B), clone 3 (C) or subclone of the clone 3 (D) for chromosomes 1 to 22.

Next, we searched for structural aberrations in the WGS data. We did not observe any gross chromosomal aberrations (Figure 3B). We observed two copy number variants (CNVs), heterozygous gains, in one of the liver organoid cultures (Figures 3C). In the other cultures, we did not detect any CNV (Figures 3D and S4B–S4D). Moreover, these two CNVs were already present in the early passage cultures and therefore did not result from long-term culturing. ES cell cultures routinely show abnormal karyotypes (Baker et al., 2007), and iPS cells can harbor considerable numbers of somatic CNVs (Hussein et al., 2011; Laurent et al., 2011; Martins-Taylor et al., 2011; Mayshar et al., 2010; Abyzov et al., 2012).

### Differentiation into Functional Hepatocytes In Vitro and upon Transplantation

**Figure 4:**
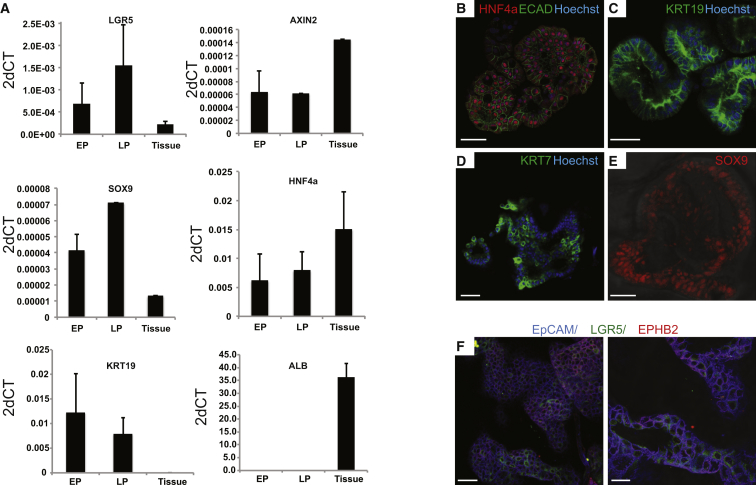
Marker Expression of Human Liver Organoids (A and B) Gene expression was analyzed by RT-PCR (A) and immunofluorescence (B) in human liver cultures grown in EM. (A) Gene expression was analyzed at early (EP) and late (LP) passages. Human liver cultures expressed progenitor (*LGR5*, *SOX9*), ductal (*KRT19*, *SOX9*), and hepatocyte (*HNF4A*) markers, but no albumin (*ALB*). Results are indicated as 2-dCt (2^ΔΔCT^). Values represent mean ±SEM of three independent experiments in five independent donor-derived cultures. 2^ΔΔCT^ were calculated using the housekeeping gene *GAPDH* as reference gene. Tissue, whole-liver lysate. (B–F) Confocal images stained for ECAD and the hepatocyte marker HNF4 (B) and the ductal markers (KRT19 [C], KRT7 [D], and SOX9 [E]). Nuclei were counterstained with Hoechst. (F) Confocal image stained for EPCAM (blue). The stem cell marker Lgr5 (green) was restricted to a subset of the cells staining for the Wnt target gene EPHB2 (red). Scale bars, 50 μm (B–E and F, left); 25 μm (F, right). See also Figure S5.

**Figure 5:**
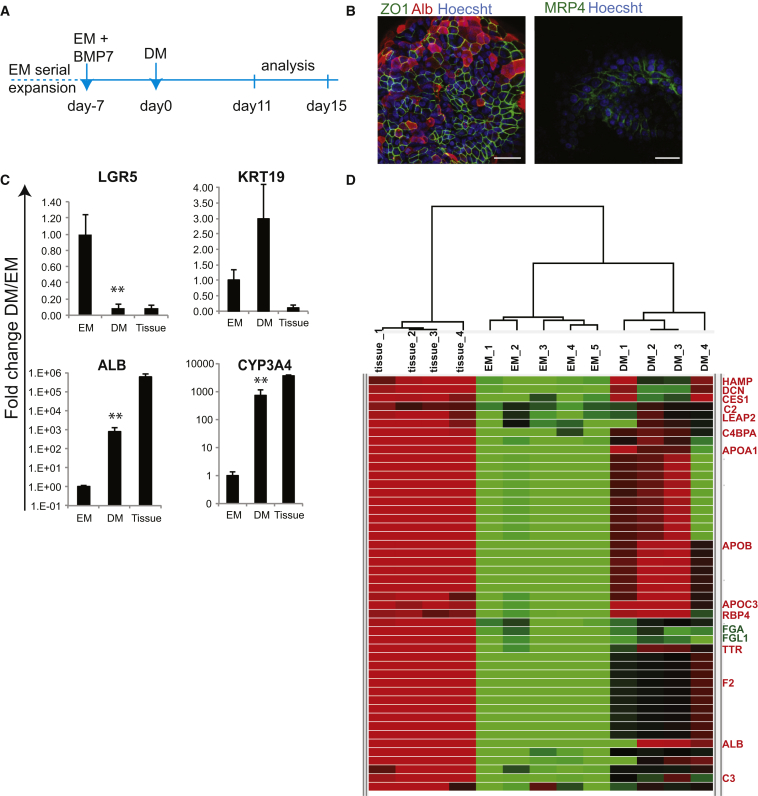
Differentiation of Organoids into Hepatocytes Human liver cultures expanded for >1 month were transferred to DM. (A) Experimental strategy. (B and C) Expression of hepatocyte genes determined by immunofluorescence (B) or qPCR (C) after 11 days. (B) Immunofluorescence for albumin (ALB, red) and ZO-1 (green). Scale bar: 25 μm, left; 30 μm, right. (C) qPCR analysis for albumin and cytochrome p450 3A4. Graphs indicate mean ±SEM of three independent experiments for three independent donors. Tissue: whole lysate from human liver. ^∗∗^p < 0.01 when comparing EM versus DM. (D) Whole-genome transcriptome analysis of human liver cultures grown in EM or after being cultured 11 days in DM. Heat map indicates cluster of genes highly expressed in liver tissue and in differentiated organoids. Of note, this cluster contains genes essential for liver function, as indicated in red. Green, downregulated; red, upregulated. See also Figure S5.

**Figure 6:**
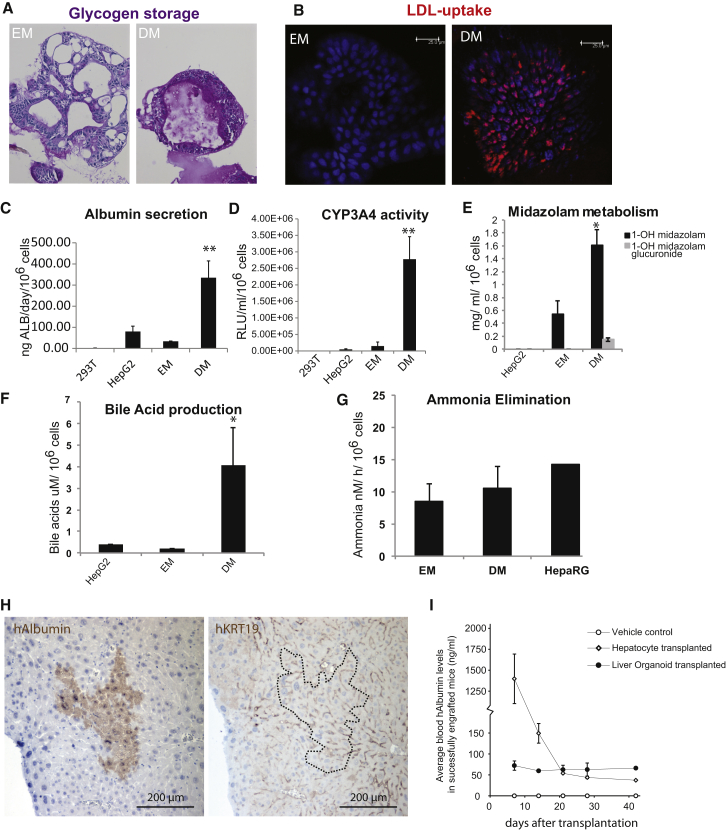
Liver Cultures Exhibit Hepatocyte Functions In Vitro and In Vivo (A) Glycogen accumulation was determined by PAS (Periodic-Acid Schiff) staining in organoids grown in EM or DM for 11 days. PAS staining (pink) was exclusively observed after differentiation (DM), indicating the capacity to accumulate glycogen. Magnification, 10×. (B) LDL uptake was analyzed using Dil-ac-LDL fluorescent substrate (red) after EM (left) or DM (right) culture for 11 days. Only cultures maintained in DM incorporated the substrate (red). Nuclei were counterstained with DRAQ5. Scale bar, 25 μm. (C) Albumin production during 24 hr was measured in supernatant. Results are expressed as mean ±SEM of two independent experiments in four independent donor-derived cultures. (D) CYP3A4 activity was measured in cultures kept in DM for 11 days. Results are expressed as RLU per ml per million cells. HEK293T cells and HepG2 cells were used as negative and positive controls, respectively. Note that DM organoids upon DM exhibit similar the CYP3A4 activity as freshly isolated hepatocytes (see Figure S2A). Triplicates for each condition were analyzed. Results are shown as mean ±SEM of two independent experiments in four independent donor-derived cultures. (E) Midazolam metabolism is performed exclusively by functional CYP3A3/4/5 enzymes. Three different organoid cultures from two different donors and HepG2 cells were cultured for 11 days as described. Midazolam was added to the medium (5 μM), and after 24 hr, concentrations of 1-OH midazolam and 1-OH midazolam glucuronide were determined. Duplicates for each condition and donor were analyzed. Results are shown as mean ±SEM of two independent experiments. (F) Bile acid production shown as ±SEM of two independent experiments in two independent donor-derived cultures. Duplicates for each condition and donor were analyzed. (G) Ammonia elimination, shown as ±SEM of n = 3 independent experiments in two independent donor-derived cultures, given as nM/h/million cells. (H) Retrorsine/CCl_4_-treated Balbc/nude mice were transplanted with 1–2 × 10^6^ human liver organoid cells and were sacrificed after 120 days. The presence of foci of human Albumin^+^/ KRT19^−^ hepatocytes demonstrates engraftment and differentiation in mouse liver. (I) Serum levels of human Albumin after transplantation. Results are shown as ±SEM of two vehicle control animals, two primary hepatocyte transplanted mice, and six human liver organoid transplanted animals. ^∗∗^p < 0.01 and ^∗^p < 0.05 when comparing EM versus DM. See also Figures S5 and S6.

**Figure S5:**
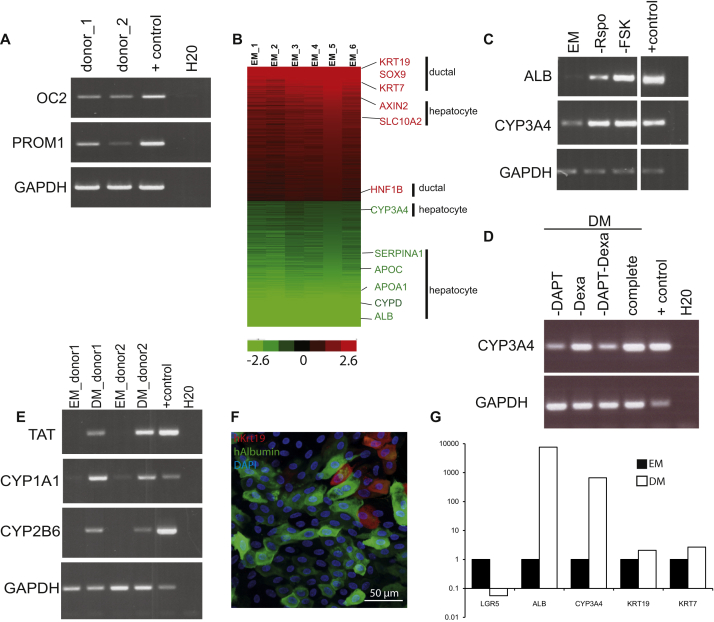
Analysis of Organoids during Expansion and upon Differentiation, Related to Figures 5 and 6 (A) Representative image of RT-PCR analysis of indicated genes in 2 independent human liver donor-derived organoid cultures maintained in Expansion medium (EM) for 2 months in culture. Note expression of progenitor marker PROM1 and ductal marker OC2 (ONECUT2). (B) Heat map of genes >2 fold differentally expressed between human liver tissue and organoid in expansion medium. Red, upregulated. Green, downregulated, Black, not differentially expressed. (C) Representative image of RT-PCR analysis of indicated genes in 1 donor derived culture maintained under complete expansion medium (EM) or after withdrawal of Rspondin (Rspo) or Forskolin (FSK). (D) Representative image of RT-PCR analysis of CYP3A4 in 1 donor derived culture maintained under complete differentiation medium (DM, complete) for 11 days, or after withdrawal of the indicated components, DAPT and/or Dexamethasone (Dexa). (E) Representative image of RT-PCR analysis of indicated genes in 2 independent human liver donor derived organoid cultures maintained in Expansion medium (EM) for 2 months in culture or after 11 days in Differentiation medium (DM). Note expression of hepatocyte markers TAT and cytochromes exclusively upon differentiation. (A-E) + control, human liver lysate (F-G) EpCAM+ cell derived organoids were differentiated for 11 days according to our differentiation protocol. (F) Immunofluorescent Albumin and Krt19 staining show presence of differentiated cells of biliary and hepatocyte lineage. (G) qRT-PCR for differentiation markers of the hepatocyte (Alb and Cyp3A) and ductal (Krt19 and Krt7) lineage show successful differentiation of EpCAM+ cell derived organoids to hepatocytes.

The stem cell markers *PROM1* and *LGR5*, as well as ductal (*SOX9*, *OC2*) and hepatocyte markers (*HNF4a*) were readily expressed (Figures 4A, S5A, and S5B). Histologically, liver organoids displayed a duct-like phenotype presenting either as: (1) a single-layered epithelium, expressing the cytokeratin markers *KRT19* and *KRT7*, or (2) a pseudo-stratified epithelium with nonpolarized E-Cadherin^+^ HNF4a+ and some KRT7+ cells (Figures 4B–4D). SOX9 (Figure 4E) and EPHB2 (Figure 4F) were detectable in almost all cells, whereas LGR5 was detectable within the EPHB2+ population (Figure 4F). The organoids failed to express markers of mature hepatocytes, such as Albumin or CYP3A4 (Figures 4A and 5C, EM bars). Therefore, we defined a human differentiation medium (DM) (Table S1). Removal of the growth stimuli R-spo and FSK resulted in upregulation of Albumin and CYP3A4 (Figure S5C). To this medium, we then added the Notch inhibitor DAPT (Huch et al., 2013b), FGF19 (Wu et al., 2011), and dexamethasone (Rashid et al., 2010) (Figure S5D). BMP7 reportedly accelerates hepatocyte proliferation in vivo (Sugimoto et al., 2007). Addition of BMP7 slightly facilitated the expression of hepatocyte markers *ALB* and *CYP3A4* even during expansion medium (data not shown). Therefore, 5–7 days prior to the start of differentiation, we added 25 ng/ml BMP7 to the expansion medium (EM) (Figure 5A). When cultured in this differentiation medium (DM), the cells acquired pronounced hepatocyte morphologies, including polygonal cell shapes (Figure 5B). Gene expression profiles revealed high levels of hepatocyte markers such as *ALB*, cytochromes, Apolipoproteins (*APOB*), and complement factors (*C3*) (Figures 5C, 5D, and S5E). Cells with high levels of ALB and MRP4 were detected by immunofluorescence (Figure 5B). Similar results were obtained with cultures derived from EpCAM^+^-sorted ductal cells (Figures S5F and S5G). Immunohistochemical analysis indicated that the cells accumulate glycogen (Figure 6A) and take up LDL (Figure 6B). Albumin was secreted into the medium (Figure 6C). The cultures exhibited similar CYP3A4 activity as fresh isolated hepatocytes (Figure 6D, compare to Figure S2A). Differentiated organoids hydroxylated midazolam, another indication of functional CYP3A3/4/5 activity (Wandel et al., 1994), and glucuronidated hydroxy-midazolam, thereby showing evidence of both phase I and II detoxifying reactions (Figure 6E). Bile acid salts were readily secreted into the medium (Figure 6F). Finally, the organoids detoxified ammonia at similar levels to HepaRG cells (Figure 6G). In all cases, the expanded human liver organoids showed stronger hepatocyte functions when compared to the standard/reference cell line HepG2 cells (Figure 6).

**Figure S6:**
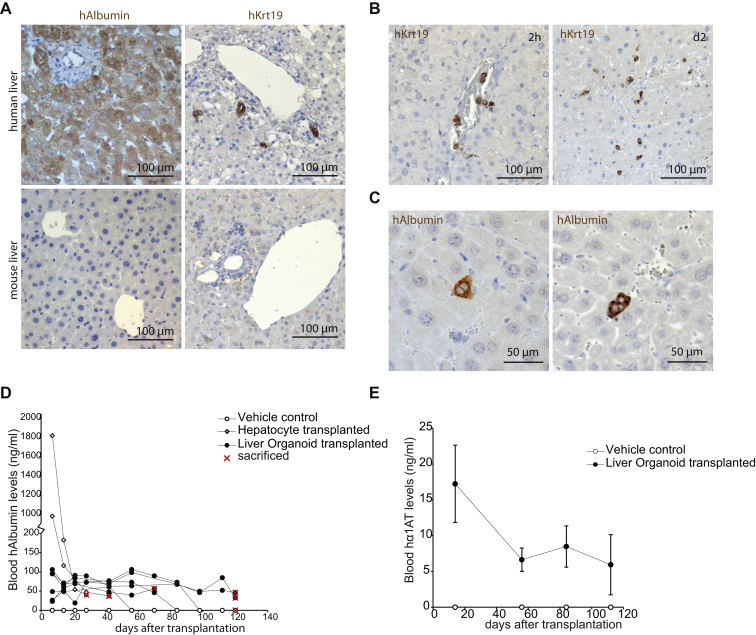
Transplantation of Human Liver Organoids into Damaged Mouse Liver, Related to Figure 6 (A) Control staining for human specific Albumin (hAlbumin) and Kertatin-19 (hKrt19) antibodies. hAlbumin recognises human but not mouse hepatocytes, whereas hKrt19 stains human but not mouse bile ducts. (B) Liver sections of mice sacrificed 2 hours or 2 days after human liver organoid cell transplantation stained for hKrt19. After 2 hours human cells are mostly seen in blood vessels in and around portal veins, whereas cells start to engraft in the tissue 2 days after the transplant. (C) Example singlet or doublet human Albumin positive hepatocytes observed in the liver of human liver organoid transplanted Balbc/nude mice. (D) Human serum Albumin levels of individual transplanted mice over 120 days. (E) Average human serum alpha-1-antitrypsin levels of transplanted mice over 120 days. Results are shown as Mean ± SEM of 2 vehicle control animals and 3 human liver organoid transplanted animals.

To test the ability of the organoids to engraft as functional hepatocytes in vivo, we treated Balb/c nude mice with CCl4-retrorsine to induce acute liver damage. This treatment allows engraftment of hepatocytes (Guo et al., 2002; Schmelzer et al., 2007). Using human-specific antibodies (Figure S6A), we initially detected *KRT19*-positive, ductal-like cells at 2 hr and 2 days after transplantation, distributed throughout the liver parenchyma (Figure S6B). At later time points, we observed *ALB*^*+*^, *KRT19*^−^ human cells as singlets/doublets or, more rarely, in larger hepatocyte foci (Figures 6H and S6C). Of note, our damage model provides no stimulus for expansion of the transplant after engraftment. Human Albumin and α-1-antitrypsin were found in serum of recipient mice within 7–14 days (Figures 6I, S6D, and S6E) at a level that remained stable for more than 60 days in five out of six mice and for more than 120 days in two out of five animals. Although transplantation of primary human hepatocytes initially yielded higher levels of human Albumin (Figure 6I), the levels approximated those of transplanted organoids within a month.

### Patient Organoids Model Disease Pathogenesis

**Figure 7:**
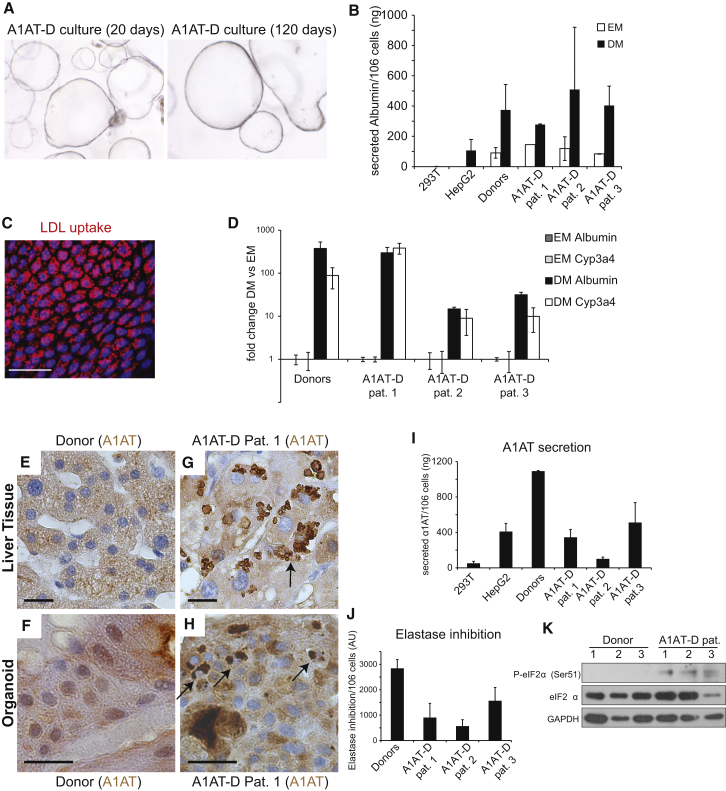
Human A1AT Deficiency Liver Cultures as an In Vitro Disease Model (A) A1AT-deficieny patient-derived liver organoids at passage 2 and passage 11 (4× magnification). (B) Albumin secretion in supernatant from donor and A1AT-deficient patient organoids in EM or after 11 days in DM. Results are expressed as mean ±SEM of two independent experiments. (C) A1AT-deficient organoids were differentiated for 11 days and incubated with DiI-Ac-LDL. Fluorescence microscopy shows robust LDL uptake in patient organoids. Scale bar, 50 μm. (D) Fold induction of Albumin and *CYP3A4* mRNA levels after 11 days of differentiation of donor and A1AT-deficient organoids. Results are expressed as mean ±SEM of two independent experiments. (E–H) Immunohistochemistry for A1AT on liver tissue (E and G) and liver-derived organoids from a healthy donor (F) and a representative A1AT deficiency patient (H) Arrows indicate A1AT protein aggregates in patient-derived liver tissue (G) and organoids (H). Scale bar, 20 μm. (I) ELISA measurement of A1AT secretion in supernatants from donor and patient organoids after 11 days of differentiation. Results are expressed as mean ±SEM of two independent experiments. (J) Enzymatic measurement of elastase inhibition by supernatants of differentiated donor and patient-derived organoids. Results are expressed as mean ±SEM of two independent experiments. (K) Western blot of lysates from donor and A1AT deficiency patient organoids after 11 days of differentiation. Increased eIF2α phosphorylation at Ser51 was detected in the three patients. Representative image is shown. Pat, patient. See also Figure S7.

**Figure S7:**
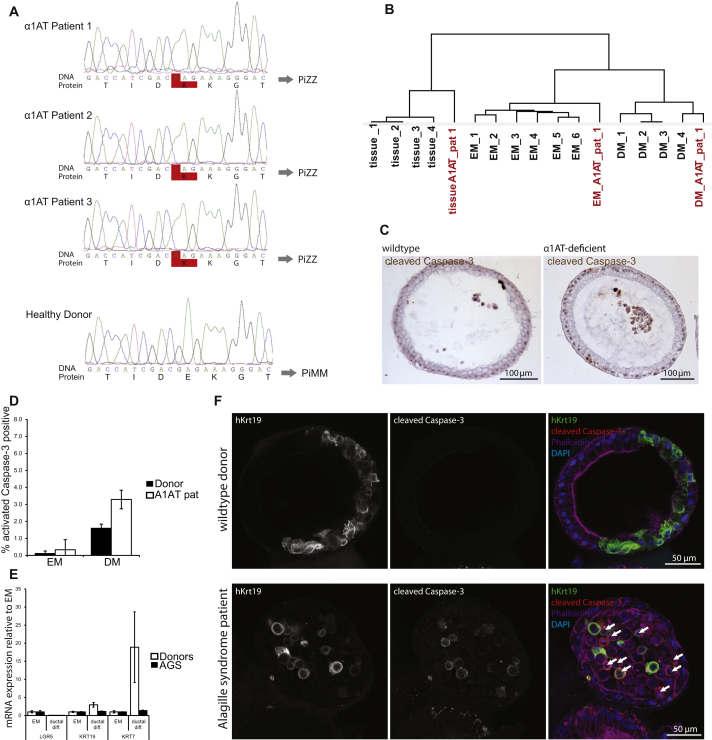
Organoids from A1AT Deficiency and AGS Patients Mimic Disease Phenotypes In Vitro, Related to Figure 7 (A) SERPIN1A Sanger Sequencing of Donor #1 and α1AT Patient #1. Chromatograms of 3 A1AT-deficient patients (PiZZ) and 1 donor with wildtype SERPINA1 (PiMM). The homozygous G to A mutation causes an amino acid change from glutamic acid to lysine at position 342. (B) Clustering analysis of the different donors (1-5) and α1AT Patient (A1AT_pat) organoids and tissues. Note that, regarding differentiation ability, the behaviour of α1AT Patient derived organoids resembles donor derived organoids. i.e. organoids in EM cluster cluster with donor EM organoids and α1AT-D organoids cultured in DM cluster with donor derived organoids cultured in DM conditions. (C) histological staining for cleaved caspase-3 in donor and α1AT Patient derived organoids differentiated in DM for 11 days. (D) quantification of apoptotic cells in wildtype and α1AT Patient derived organoids in EM and after differentiation in DM. Results are shown as Mean ± SEM of 6 random sections of organoids per 2 independent donors and patients. (E) qRT-PCR of Lgr5 and ductal markers (Krt19 and Krt7) in EM and after ductal differentiation. AGS patients fail to upregulate ductal markers upon differentiation. Results are shown as Mean ± SEM of 3 independent experiments. (F) Immunofluorescence of differentiated wildtype and AGS patient organoids. Krt19 positive cells in AGS patient organoids do not integrate into the epithelium and show signs of apoptosis (arrows). EM, expansion medium. DM, differentiation medium, ductal diff, ductal differentiation medium (see text). AGS, Alagille syndrome.

α1-antitrypsin (A1AT) deficiency is an inherited disorder that predisposes to chronic obstructive pulmonary disease and chronic liver disease (Stoller and Aboussouan, 2005). A1AT is secreted from the liver to protect the lung against proteolytic damage from neutrophil elastase. The most frequent mutation is the Z allele (Glu342Lys) of the *SERPINA1* gene, which causes accumulation of misfolded A1AT in hepatocytes. The ZZ mutant phenotype is characterized by a ∼80% reduction of the protein in plasma, which subsequently causes lung emphysema (Stoller and Aboussouan, 2005). Biopsies from three patients diagnosed with A1AT deficiency (Table S2 and Figure S7A) were subjected to histological characterization, RNA, and DNA isolation and expansion in culture. Organoids were grown for >4 months in culture and behaved normally. Gene expression analysis demonstrated that the cells differentiated normally in DM (Figure S7B). Functional tests revealed that the differentiated cells from A1AT patients secreted high levels of Albumin and take up LDL similar to that of healthy donor-derived organoid cultures (Figures 7B–7D). In A1AT deficiency, the molecular pathogenesis of the liver disease relates to the aggregation of the protein within the endoplasmic reticulum of hepatocytes (Lawless et al., 2008). A1AT protein aggregates were readily observed within the cells of the differentiated organoids (Figure 7H), similar to what was found in the original biopsy (Figure 7G). A1AT ELISA confirmed reduced protein secretion (Figure 7I) (Table S2 indicates the A1AT secretion per patient), and supernatants from differentiated mutant organoids showed reduced ability to block elastase activity (Figure 7J). Protein misfolding is one of the primary causes that drive hepatocytes apoptosis in PiZZ individuals (Lawless et al., 2008). Differentiated liver organoids from A1AT-D patients mimicked the in vivo situation and showed signs of ER stress, such as phosphorylation of eIF2α (Figure 7K) and increased apoptosis in the differentiated state (Figures S7C and S7D).

Using a biopsy from an Alagille syndrome (AGS) patient, we tested whether structural defects of the biliary tree can also be modeled. AGS is caused by mutations in the Notch-signaling pathway, which results in partial to complete biliary atresia (Kamath et al., 2013). Patient organoids resembled their healthy counterparts in the undifferentiated state. However, upon differentiation to the biliary fate by withdrawal of R-spondin, Nicotinamide, TGFbi, and FSK, AGS patient organoids failed to upregulate biliary markers such as *KRT19* and *KRT7* (Figure S7E). Staining for KRT19 revealed that biliary cells were scarce and unable to integrate into the epithelium. Rather, they rounded up and underwent apoptosis in the organoid lumen (Figure S7F). In AGS mouse models, *JAGGED-1/NOTCH2* is dispensable for biliary lineage specification but is required for biliary morphogenesis (Geisler et al., 2008; McCright et al., 2002). Thus, AGS liver organoids constitute the first human 3D model system to study Alagille syndrome.

## Discussion

Liver diseases (ranging from genetic inherited disorders to viral hepatitis, liver cancer, and obesity-related fatty liver disease) account for the twelfth-leading cause of death in the United States (Heron, 2012). Failure in the management of liver diseases can be attributed to the shortage of donor livers (Vilarinho and Lifton, 2012) as well as to our poor understanding of the mechanisms behind liver pathology. The value of any cultured cell as a disease model or as a source for cell therapy transplantation depends on the fidelity and robustness of its expansion potential as well as its ability to maintain a normal genetic and epigenetic status (Pera, 2011). The possibility of differentiating hESC or reprogrammed fibroblasts (iPS) into almost any differentiated cell type, from neurons to hepatocytes, has allowed modeling of many human genetic diseases, including A1AT-D (Rashid et al., 2010). However, the genetic instability of cultured stem cells raises concerns regarding their safe use in cell therapy transplantation (Bayart and Cohen-Haguenauer, 2013).

Here, we show that primary human bile duct cells can readily be expanded in vitro as bipotent stem cells into 3D organoids. These cells differentiate into functional hepatocyte cells in vitro and generate bona fide hepatocytes upon transplantation. Extensive analysis of the genetic stability of cultured organoids in vitro demonstrates that the expanded cells preserve their genetic integrity over months in culture. These results agree with our previous observations in the mouse (Huch et al., 2013b) yet are in striking contrast to recent publications in which, utilizing several lineage tracing approaches, ductal/resident stem cells have been described as not contributing to mouse liver regeneration (Schaub et al., 2014; Yanger et al., 2014; Yanger et al., 2013). Our results resemble what has been elegantly shown in zebrafish and rat models: in the event of an almost complete hepatocyte loss or blockage of hepatocyte proliferation, biliary epithelial cells convert into hepatocytes (Choi et al., 2014) (Michalopoulos, 2014). Our data are further corroborated in human fulminant hepatic failure, in which, upon 80% loss of hepatocyte compartment, huge numbers of proliferating EpCAM+ biliary epithelial cells are observed (Hattoum et al., 2013).

Organoids from A1AT-deficiency patients can be expanded in vitro and mimic the in vivo pathology. Similarly, organoids from an Alagille syndrome patient reproduce the structural duct defects present in the biliary tree of these patients. Repair by homologous recombination using CRISPR/Cas9 technology is feasible in organoid cultures, as we recently demonstrated in colon stem cells of cystic fibrosis patients (Schwank et al., 2013). A variety of monogenic hereditary diseases affect the liver specifically, and these should all be amenable to a comparable in vitro approach of gene repair in clonal liver progenitor cells. Overall, our results open up the avenue to start testing human liver material expanded in vitro as an alternative cell source for studies of human liver regeneration, human liver disease mechanism, cell therapy transplantation, toxicology studies, or drug testing.

## Experimental Procedures

### Human Liver Organoid Culture

Liver biopsies (0.5–1 cm^3^) were obtained from donor and explant livers during liver transplantation performed at the Erasmus MC, Rotterdam. The Medical Ethical Council of the Erasmus Medical Center approved the use of this material for research purposes, and informed consent was provided from all patients. For EpCAM sorting experiments and hepatocyte isolation, primary human liver tissue was obtained with informed consent and approval by the Regional Ethics Board, from the CLINTEC division of Karolinska institute (Dnr: 2010/678-31/3) (Jorns et al., 2014). Liver cells were isolated from human liver biopsies (0.5–1 cm^3^) by collagenase-accutase digestion, as described in the Extended Experimental Procedures. The different fractions were mixed and washed with cold Advanced DMEM/F12 and spun at 300–400 × *g* for 5 min. The cell pellet was mixed with Matrigel (BD Biosciences) or reduced growth factor BME 2 (Basement Membrane Extract, Type 2, Pathclear), and 3,000–10,000 cells were seeded per well in a 48-well/plate. Non-attaching plates were used (Greiner). After Matrigel or BME had solidified, culture medium was added. Culture media was based on AdDMEM/F12 (Invitrogen) supplemented with 1% N2 and 1% B27 (both from GIBCO), 1.25 mM N-Acetylcysteine (Sigma), 10 nM gastrin (Sigma), and the growth factors: 50 ng/ml EGF (Peprotech), 10% RSPO1 conditioned media (homemade), 100 ng/ml FGF10 (Peprotech), 25 ng/ml HGF (Peprotech), 10 mM Nicotinamide (Sigma), 5 uM A83.01 (Tocris), and 10 uM FSK (Tocris). For the establishment of the culture, the first 3 days after isolation, the medium was supplemented with 25 ng/ml Noggin (Peprotech), 30% Wnt CM (homemade prepared as described in Barker et al. [2010]), and 10 uM (Y27632, Sigma Aldrich) or hES cell cloning recovery solution (Stemgent). Then, the medium was changed into a medium without Noggin, Wnt, Y27632, hES cell cloning recovery solution. After 10–14 days, organoids were removed from the Matrigel or BME, mechanically dissociated into small fragments, and transferred to fresh matrix. Passage was performed in a 1:4–1:8 split ratio once every 7–10 days for at least 6 months. To prepare frozen stocks, organoid cultures were dissociated and mixed with recovery cell culture freezing medium (GIBCO) and frozen following standard procedures. When required, the cultures were thawed using standard thawing procedures and cultured as described above. For the first 3 days after thawing, the culture medium was supplemented with Y-27632 (10 μM).

Growth curves and expansion ratios were performed and calculated as described in the Extended Experimental Procedures.

### Isolation of EpCAM+ Cells and Single-Cell Culture

Cell suspensions prepared as described in the Extended Experimental Procedures were stained with anti-human CD326 (EpCAM), sorted on a MoFlo (Dako Cytomation) sorter, and cultured as described above with medium supplemented with Y-27632 (10 μM, Sigma Aldrich) for the first 4 days. Passage was performed in split ratios of 1:4–1:8 once per week.

For clonogenic assays, single-cell suspensions were sorted using FSC and pulse width to discriminate single cells. Propidium iodide staining was used to label dead cells and FSC: pulse-width gating to exclude cell doublets (MoFlow, Dako). Sorted cells were embedded in Matrigel and seeded in 96-well plates at a ratio of 1 cell/well. Cells were cultured as described above.

### Hepatocyte Differentiation and In Vitro Functional Studies

Liver organoids were seeded and kept 7–10 days under the liver medium explained above (EM, expansion medium) supplemented with BMP7 (25 ng/ml). Then, the cultures were split and seeded accordingly in this EM supplemented with BMP7 for at least 2–4 days. Then, medium was changed to the differentiation medium (DM): AdDMEM/F12 medium supplemented with 1% N2 and 1% B27 and containing EGF (50 ng/ml), gastrin (10 nM, Sigma), HGF (25 ng/ml), FGF19 (100 ng/ml), A8301 (500 nM), DAPT (10 uM), BMP7 (25 ng/ml), and dexamethasone (30 uM). Differentiation medium was changed every 2–3 for a period of 11–13 days.

To assess hepatocyte function, culture medium was collected 24 hr after the last medium change. Functional studies were performed in the collected supernatant or in whole organoids, as described in the Extended Experimental Procedures.

### Transplantation

We used a modified version of the protocol used by Guo et al. (Guo et al., 2002). In short, female BALB/c nude mice (around 7 weeks of age) were pretreated with two injections of 70 mg/kg Retrorsine (Sigma) at 30 and 14 days before transplantation. One day prior to transplantation, mice received 0.5 ml/kg CCl4 and 50 mg/animal anti-asialo GM1 (Wako Pure Chemical Industries) via IP injection. Furthermore, animals received 7.5 ug/ml FK506 in drinking water until the end of the experiment due to the reported positive effects on liver regeneration (He et al., 2010). On the day of transplantation, mice were anaesthetized, and suspensions of 1–2 × 10^6^ human liver organoid cells derived from four independent donors (p6–p10) or fresh isolated hepatocytes (two donors) were injected intrasplenically. Transplanted mice received weekly injections of 50 mg/animal anti-asialo GM1 (Wako Pure Chemical Industries) to deplete NK cells. To monitor the transplantation state, blood samples were taken in regular intervals from the tail vein and were analyzed for the presence of human albumin and human α1-antitrypsin using respective human specific ELISAs (Assaypro).

### Karyotyping and Genetic Stability Analysis

Organoid cultures in exponential growing phase were incubated for 16 hr with 0.05 μg/ml colcemid (GIBCO). Then, cultures were dissociated into single cells using TrypLE express (GIBCO) and processed using standard karyotyping protocols.

DNA libraries for WGS analysis were generated from 1 μg of genomic DNA using standard protocols (Illumina). The libraries were sequenced with paired-end (2 × 100 bp) runs using Illumina HiSeq 2500 sequencers to a minimal depth of 30× base coverage (average depth of ∼36.9× base coverage). As a reference sample, liver biopsies was sequenced to equal depth for the different donors. Analysis of the sequence reads, calling of CNVs, and base substitutions are described in detail in the Extended Experimental Procedures. The data for the whole-genome sequencing were deposited to the EMBL European Nucleotide Archive with accession number ERP005929.

### Immunohistochemistry, Immunofluorescence, and Image Analysis

Tissues and organoids were fixed o/n with formalin or 4% PFA, respectively, and stained and imaged as described in the Extended Experimental Procedures.

### A1AT-D Functional Experiments

Elastase inhibition assay and detection of phosphorylated eIF2α were performed as described in the Extended Experimental Procedures.

### Microarray

For the expression analysis of human liver cultures, total RNA was isolated from liver biopsies or from organoid cultures grown in our defined medium, using QIAGEN RNAase kit following the manufacturer’s instructions. Five hundred ng of total RNA were labeled with low RNA Input Linear Amp kit (Agilent Technologies). Universal human reference RNA (Agilent) was differentially labeled and hybridized to the tissue or cultured samples. A 4X 44 K Agilent whole human genome dual color microarray (G4122F) was used. Labeling, hybridization, and washing were performed according to Agilent guidelines. Microarray signal and background information were retrieved using Feature Extraction software (V.9.5.3, Agilent Technologies). Hierarchical clustering analysis was performed in whole-liver tissue or organoid arrays. A cut-off of 3-fold differentially expressed was used for the clustering analysis.

### Data Analysis

All values are represented as mean ± SEM. Man-Whitney nonparametric test was used. p < 0.05 was considered statistically significant. In all cases, data from at least three independent experiments was used. All calculations were performed using SPSS package.

Extended Experimental Procedures **Human Liver Isolation**

Liver cells were isolated by collagenase digestion as follows: tissue (0.5-1cm3) was minced, rinsed 2x with DMEM (GIBCO) 1%FCS and incubated with the digestion solution (2.5 mg/ml collagenase D (Roche) + 0.1 mg/ml DNase I (Sigma) in EBSS (Hyclone, Thermoscientific), for 20-40 at 37°C. The digestion was stopped by adding cold DMEM 1%FCS and the suspension was then filtered through a 70 um Nylon cell strainer and spun 5 min at 300-400 g. The pellet was resuspended in DMEM 1%FCS and kept cold. Any material retained on the strainer was further digested for 10 min in Accutase (GIBCO) at 37C. Then, the digestion was stopped and the cells were collected as before. The different fractions (collagenase and accutase) were seeded and cultured as described in Experimental Procedures.

**In Vitro Growth Curves**

Expansion ratios were calculated from human liver cultures as follows: 3x10^3^ cells were grown in our defined medium for 7 or 10 days. Then, the cultures were dissociated by incubation with TrypLE Express (GIBCO) until single cells. Cell numbers were counted by trypan blue exclusion at the indicated time points. From the basic formula of the exponential curve *y(t)* = *y*_*0*_ x *e*^*(growth rate x t)*^ (*y* = cell numbers at final time point; *y*_*0*_ = cell numbers at initial time point; *t* = time) we derived the growth rate. Then, the doubling time was calculated as doubling time = ln(2)/growth rate for each time window analyzed.

**Isolation of EpCAM+ Cells from Primary Human Liver**

Human liver cells were isolated according to standard protocol at the liver cell laboratory at the unit for transplantation surgery, CLINTEC, Karolinska Institute (Jorns et al., 2014). Cell suspensions were shipped overnight on ice. Suspensions were diluted in 2 volumes of cold Advanced DMEM/F12 (GIBCO) and washed 3 times in the same medium. Viable cells were counted with Trypan blue and split into 3 parts for EpCAM sorting, Percoll purification (see Hepatocyte Percoll purification) and direct seeding into matrigel. For sorting, liver cells were stained with 1:100 Anti-Human CD326 (EpCAM) Alexa Fluor® 488 (eBioscience) for 30 min at 4°C. Subsequently cells were washed and sorted on a MoFlo (Dako Cytomation) cell sorter. Sorted cells were spun down, resuspended in Matrigel and grown into organoids according to standard human liver organoid culture procedure (see Human liver organoid culture). After 14 days in culture the number of organoids larger than 100 μm in diameter was scored.

**Hepatocyte Percoll Purification and Cyp3a4 Measurement**

Human hepatocyte suspensions (see Isolation of EpCAM+ cell from primary human liver) were washed as described, spun down and resuspended in 35 ml Advanced DMEM/F12 (GIBCO) + 13.5 ml Percoll (GE healthcare, density 1.130 g/ml) + 1.5 ml 10x HBSS (GIBCO). Cells were pelleted at 100 g for 10 min and washed 3 times in Advanced DMEM/F12 (GIBCO). Viable cells were counted with Trypan blue and 10.000 viable cells per 50 ul drop were seeded into matrigel. Remaining cells were stained for EpCAM as described above (see Isolation of EpCAM+ cells from primary human liver) or seeded onto collagen coated tissue culture plates for subsequent determination of cytochrome 3A4 activity. To measure Cyp3a4 in primary hepatocytes, the seeded cells were cultured in Williams E medium (GIBCO) containing Hepatocyte plating supplement pack (GIBCO) for 4 days with daily medium changes. On day 0 and day 4 the cells were incubated with Luciferin-PFBE substrate (50 μM) and Cytochrome P450 activity was measured using the P450-Glo Assay Kit (Promega) according to manufacturer’s instructions and normalized to the number of cells in the plate. HepG2 cells cultured in the same medium served as controls.

**Genetic Analysis**

DNA libraries for WGS analysis were generated from 1 μg of genomic DNA using standard protocols (Illumina). The libraries were sequenced with paired-end (2 × 100 bp) runs using Illumina HiSeq 2500 sequencers to a minimal depth of 30 x base coverage (average depth of ∼36.9 x base coverage). As reference sample, liver biopsies was sequenced to equal depth for the different donors. Sequence reads were mapped against human reference genome GRCh37 using Burrows-Wheeler Aligner (BWA) 0.7.5a with settings ‘bwa mem -c 100 -M’ resulting in sample-specific BAM files. To predict CNVs, BAM files were analyzed using Control-FREEC (Boeva et al., 2012) and DELLY (Rausch et al., 2012). To obtain somatically acquired CNVs, we filtered called CNVs for occurrence in the reference samples (liver biopsies). Single nucleotide variants (SNVs) were multi-sampled called using the Genome Analysis Toolkit (GATK) v2.7.2 UnifiedGenotyper (DePristo et al., 2011). We only considered positions at autosomal chromosomes, which were covered at least 20x in all liver stem cell samples and corresponding biopsy from the same donor. Candidate somatic SNVs were further filtered using the following criteria: no evidence in reference samples; minimal alternative allele frequency of 0.3 to exclude sequencing artifacts and potential substitutions that occurred after the clonal step; a minimal GATK quality score of 100; no overlap with single nucleotide polymorphisms (SNPs) in the Single Nucleotide Polymorphism Database (dbSNP 137.b37); and no overlap with SNVs in the other tested individual (Figure S2). Ultimately, SNVs with evidence in both clonal and subclonal cultures were considered as in vivo acquired somatic variation, and SNVs with evidence in only subclonal cultures were considered as variation accumulated during in vitro culturing.

**Immunohistochemistry, Immunofluorescence, and Image Analysis**

Tissues and organoids were fixed o/n with formalin or 4% PFA respectively, and stained washed and transferred to tissue cassettes and paraffin blocks using standard methods. Tissue sections (4 μM) were prepared and stained with antibodies, H&E or PAS using standard techniques. The antibodies and dilutions used are listed in Table S5. Stained tissues were counterstained with Mayer’s Hematoxylin. Pictures were taken with a Nikon E600 camera and a Leica DFDC500 microscope (Leica). For whole mount immunofluorescence staining, organoids were processed as described in Barker et al., (Barker et al., 2010). Nuclei were stained with Hoechst33342 (Molecular Probes). Immunofluorescence images were acquired using a confocal microscope (Leica, SP5). Images were analyzed and processed using Leica LAS AF Lite software (Leica SP5 confocal). All phase contrast pictures were acquired using a Leica DMIL microscope and a DFC420C camera.

**RT-PCR and qPCR Analysis**

RNA was extracted from organoid cultures or freshly isolated tissue using the RNeasy Mini RNA Extraction Kit (QIAGEN), and reverse-transcribed using reverse-transcribed using Moloney Murine Leukemia Virus reverse transcriptase (Promega). All targets were amplified (40 cycles) using gene-specific primers and MiIQ syber green (Bio-Rad). Data were analyzed using BioRad CFX manager. For Figure S7, cDNA was amplified in a thermal cycler (GeneAmp PCR System 9700; Applied Biosystems, London, UK) as previously described (Huch et al., 2009). Primers used are listed in Table S4.

**Functional Hepatocyte Studies**

To assess glycogen storage and LDL uptake, liver organoids grown in EM or DM for 11 days were stained by Periodic acid-Schiff (PAS, Sigma) and DiI-Ac-LDL (Biomedical Technologies), respectively, following manufacturer’s instructions. To determine albumin and A1AT secretion, liver organoids were differentiated as described. Culture medium was changed every 3-4 days and culture supernatant was collected was collected 24h after the last medium change. HepG2 (ATCC number 77400) and HEK293T (ATCC number CRL-3216) cells were cultured for 24h in the same medium without growth factors and were used as positive and negative control respectively. The amount of albumin and A1AT in culture supernatant was determined using a human specific Albumin or human specific A1AT ELISA kit (both from Assay Pro). To measure Cyp3a activity the cultures were differentiated as described and the day of the experiment the cells were removed from the matrigel and cultured with the Luciferin-PFBE substrate (50 μM) in Hepatozyme medium supplemented with 10% FBS (GIBCO). As controls, HepG2 and HEK293Tcells were cultured for 24h in DMEM 10%FBS and the day of the experiment transferred to Hepatozyme medium supplemented with 10% FBS (GIBCO) and Luciferin-PFBE substrate (50 μM). Cytochrome P450 activity was measured 8h later using the P450-Glo Assay Kit (Promega) according to manufacturer’s instructions.

Concentrations of midazolam, 1-hydroxymidazolam (1-OH-M) and 1-hydroxymidazolam-glucuronide (1-OH-MG) were determined in 50 μl using LC-MS/MS. Analysis was carried out at the Clinical Pharmaceutical and Toxicological Laboratory of the Department of Clinical Pharmacy of the University Medical Center Utrecht, the Netherlands. All experiments were performed on a Thermo Fisher Scientific (Waltham, MA) triple quadrupole Quantum Access LC-MS/MS system with a Surveyor MS pump and a Surveyor Plus autosampler with an integrated column oven. Analytes were detected via MS/MS, with an electrospray ionization-interface in selected reaction monitoring-mode, by their parent and product ions. The method showed linearity over the range of 0.02 – 1.50 mg/L for MDZ and OHM and over the range of 0.10 – 10.0 mg/L for HMG. The analytical accuracy and precision were within the maximum tolerated bias and CV (20% for LLOQ, 15% for the other concentrations). Since a 1-OH-MG standard was not available, a Gold Standard was used. The Gold Standard consisted of the urine from two adult intensive care patients with a high dose of intravenous midazolam and good renal function. Total bile acids were measured on an AU5811 routine chemistry analyzer (Beckman Coulter, Brea, California) with an enzymatic colorimetric assay (Sentinel Diagnostics, Milano, Italy). Ammonia elimination was analyzed as follows: organoid cultures were expanded and differentiated in DM medium for 8 days. On day 8 CAG was added to the medium and 3 days later the organoids were remouved from the matrigel, washed with Williams’ medium and subsequently incubated with 1 ml of test medium (Williams’ E medium (Lonza, Basel, Switzerland) with 10% fetal bovine serum (Lonza), 5 μg / mL insulin (Sigma, St. Louis, U.S.), 50 μM hydrocortisone hemisuccinate (Sigma), 2mM glutamine (Lonza), 50 U / mL penicilline and 50 μg / mL streptomycin (penicilline/streptomycine mix (Lonza), 1.5 mM NH4Cl (Sigma), 2.27 mM D-galactose (Sigma), 2 mM L-lactate (Sigma) and 2 mM ornithine hydrochloride (Sigma)). Then 0.25 ml samples were taken after 45 min, 7 and 24 hr and stored at −20°C for further analysis. Subsequently, all cultures were washed twice with PBS, trypsinized and cell number was counted by tripan blue exclusion.

Concentrations of ammonia were assessed in all samples by using the Ammonia (rapid) kit (Megazyme International, Wicklow, Ireland). The rates of ammonia elimination were established by calculating the changes in absolute molecular amounts of ammonia in the medium and corrected for time and cell number.

**SERPINA1 Sequencing**

All 4 SERPINA1 exons were amplified from genomic DNA using Phusion High-fidelity DNA polymerase (Thermo Scientific) and specific primersets (see Table S4). PCR products were purified using QIAquick PCR purification kit (QIAGEN) and sequenced on an ABI 3730XL capillary sequencer.

**Enzymatic Elastase Inhibition Assay**

For measurement of the inhibitory action of α1-antitrypsin in organoid supernatants, donor and patient organoids were differentiated for 11 days. Culture medium was changed every 2-3 days and culture supernatant was collected 24h after the last medium change. For the assay, 160 ul of supernatant are mixed with 20 ul of a 2 mg/ml N-Succinyl-Ala-Ala-Ala-p-nitroanilide (Sigma) 100 mM Tris pH 8.0 solution in a clear-bottom 96-well plate. After addition of 6x10-4 U of Elastase (porcine pancreas, Sigma) in 100 mM Tris pH 8.0, the increase in absorbance at 410 nm is measured continuously over 30 min. Elastase inhibition by supernatants is measured as the decreased inclination of absorbance over time in comparison to uninhibited controls (plain medium) and compared to a dilution series of purified human α1-antitrypsin (Zemaira) in medium.

**Detection of eIF2α Phosphorylation**

Donor and α1-antitrypsin deficient patient organoids were differentiated for 11 days. Culture medium was changed every 2-3 days and organoids were lysed in Lysis buffer (50 mM Tris pH 7.5, 50 mM NaCl, 0.5% Triton X-100, 0.5% NP40 substitute, 5 mM EGTA, 5 mM EDTA, 1x Complete protease inhibitor (Roche), 1x PhosStop (Roche)). Using standard techniques lysates were resolved by SDS-Page and blotted on PVDF membranes (Millipore). Antibodies against are listed in Table S5.

## Author Contributions

M.H., H.G., and H.C. designed and, together with K.H., performed and analyzed experiments. M.H. designed and developed and, with K.H., performed all experiments and analyzed all data that characterized the human liver culture system. M.H., R.v.B., E.C., and H.C. designed the genetic studies. M.H. and H.G. designed and M.H., H.G., and K.H. performed A1AT experiments. H.G. and H.C. designed and H.G. and K.H. performed ductal origin, transplantation, and AGS experiments. R.v.B. performed the genetic stability studies, supervised the next-gen sequencing, and set up the filtering pipeline. F.B. adjusted and applied pipeline. J.d.L. performed the CNV analysis. M.H., M.v.W., R.H., S.A.F., S.J.B., and H.K. performed functional in vitro experiments and analyzed the data. M.v.d.W. and N.S. performed FACS. M.M.A.V., J.N.M.I., S.S., E.E. and L.J.W.v.d.L. provided Ethical Aproval, human liver donor biopsies, isolated hepatocytes, and patient material. E.E.S.N. and R.R.G.V. provided METC. R.R.G.V provided helpful discussions. M.H., H.G., R.v.B., E.C., and H.C. wrote the manuscript. All authors commented on the manuscript.

## Accession Numbers

The data for the whole-genome sequencing of clonal organoid cultures has been deposited to the EMBL European Nucleotide Archive under accession number ERP005929. The gene expression data reported in this paper has been deposited at the GEO repository with accession number GSE63859.

## Supplemental Information

Table S1. List of Tested Compounds, Related to Figure 1 List of all the compounds tested for their capacity to enhance human liver culture proliferation, long-term maintenance, or differentiation. Human liver cultures were seeded in ERFHNic medium supplemented with A8301 and the compound indicated on the list. Seeding efficiency and capacity to expand long-term the cultures was evaluated. Green, the compound supports human liver growth. Red, the compound has a negative effect on the culture. Absence of color, the compound does not affect either way.

Table S2. Human Material Cultured, Related to Figures 1–7 Patient and donor information from the cultured material. When available, A1AT protein serum levels are listed

Table S3. List of Mutated Genes with Nonsynonymous Mutations Found after WGS Analysis, Related to Figure 2

Table S4. List of Primers Used, Related to Experimental Procedures

Table S5. List of Antibodies Used, Related to Experimental Procedures

Document S1. Article plus Supplemental Information

## References

[bib1] Abyzov A., Mariani J., Palejev D., Zhang Y., Haney M.S., Tomasini L., Ferrandino A.F., Rosenberg Belmaker L.A., Szekely A., Wilson M. (2012). Somatic copy number mosaicism in human skin revealed by induced pluripotent stem cells. Nature.

[bib2] Baker D.E., Harrison N.J., Maltby E., Smith K., Moore H.D., Shaw P.J., Heath P.R., Holden H., Andrews P.W. (2007). Adaptation to culture of human embryonic stem cells and oncogenesis in vivo. Nat. Biotechnol..

[bib3] Barker N., van Es J.H., Kuipers J., Kujala P., van den Born M., Cozijnsen M., Haegebarth A., Korving J., Begthel H., Peters P.J., Clevers H. (2007). Identification of stem cells in small intestine and colon by marker gene Lgr5. Nature.

[bib4] Barker N., Huch M., Kujala P., van de Wetering M., Snippert H.J., van Es J.H., Sato T., Stange D.E., Begthel H., van den Born M. (2010). Lgr5(+ve) stem cells drive self-renewal in the stomach and build long-lived gastric units in vitro. Cell Stem Cell.

[bib5] Bayart E., Cohen-Haguenauer O. (2013). Technological overview of iPS induction from human adult somatic cells. Curr. Gene Ther..

[bib6] Bigorgne A.E., Farin H.F., Lemoine R., Mahlaoui N., Lambert N., Gil M., Schulz A., Philippet P., Schlesser P., Abrahamsen T.G. (2014). TTC7A mutations disrupt intestinal epithelial apicobasal polarity. J. Clin. Invest..

[bib7] Carmon K.S., Gong X., Lin Q., Thomas A., Liu Q. (2011). R-spondins function as ligands of the orphan receptors LGR4 and LGR5 to regulate Wnt/beta-catenin signaling. Proc. Natl. Acad. Sci. USA.

[bib8] Cheng L., Hansen N.F., Zhao L., Du Y., Zou C., Donovan F.X., Chou B.K., Zhou G., Li S., Dowey S.N., NISC Comparative Sequencing Program (2012). Low incidence of DNA sequence variation in human induced pluripotent stem cells generated by nonintegrating plasmid expression. Cell Stem Cell.

[bib9] Choi T.Y., Ninov N., Stainier D.Y., Shin D. (2014). Extensive conversion of hepatic biliary epithelial cells to hepatocytes after near total loss of hepatocytes in zebrafish. Gastroenterology.

[bib10] de Lau W., Barker N., Low T.Y., Koo B.K., Li V.S., Teunissen H., Kujala P., Haegebarth A., Peters P.J., van de Wetering M. (2011). Lgr5 homologues associate with Wnt receptors and mediate R-spondin signalling. Nature.

[bib11] Dekkers J.F., Wiegerinck C.L., de Jonge H.R., Bronsveld I., Janssens H.M., de Winter-de Groot K.M., Brandsma A.M., de Jong N.W., Bijvelds M.J., Scholte B.J. (2013). A functional CFTR assay using primary cystic fibrosis intestinal organoids. Nat. Med..

[bib12] Dorrell C., Erker L., Schug J., Kopp J.L., Canaday P.S., Fox A.J., Smirnova O., Duncan A.W., Finegold M.J., Sander M. (2011). Prospective isolation of a bipotential clonogenic liver progenitor cell in adult mice. Genes Dev..

[bib13] Duncan A.W., Dorrell C., Grompe M. (2009). Stem cells and liver regeneration. Gastroenterology.

[bib14] Francis H., Glaser S., Ueno Y., Lesage G., Marucci L., Benedetti A., Taffetani S., Marzioni M., Alvaro D., Venter J. (2004). cAMP stimulates the secretory and proliferative capacity of the rat intrahepatic biliary epithelium through changes in the PKA/Src/MEK/ERK1/2 pathway. J. Hepatol..

[bib15] Furuyama K., Kawaguchi Y., Akiyama H., Horiguchi M., Kodama S., Kuhara T., Hosokawa S., Elbahrawy A., Soeda T., Koizumi M. (2011). Continuous cell supply from a Sox9-expressing progenitor zone in adult liver, exocrine pancreas and intestine. Nat. Genet..

[bib16] Geisler F., Nagl F., Mazur P.K., Lee M., Zimber-Strobl U., Strobl L.J., Radtke F., Schmid R.M., Siveke J.T. (2008). Liver-specific inactivation of Notch2, but not Notch1, compromises intrahepatic bile duct development in mice. Hepatology.

[bib17] Gore A., Li Z., Fung H.L., Young J.E., Agarwal S., Antosiewicz-Bourget J., Canto I., Giorgetti A., Israel M.A., Kiskinis E. (2011). Somatic coding mutations in human induced pluripotent stem cells. Nature.

[bib18] Gramignoli R., Green M.L., Tahan V., Dorko K., Skvorak K.J., Marongiu F., Zao W., Venkataramanan R., Ellis E.C., Geller D. (2012). Development and application of purified tissue dissociation enzyme mixtures for human hepatocyte isolation. Cell Transplant..

[bib19] Guo D., Fu T., Nelson J.A., Superina R.A., Soriano H.E. (2002). Liver repopulation after cell transplantation in mice treated with retrorsine and carbon tetrachloride. Transplantation.

[bib20] Hattoum A., Rubin E., Orr A., Michalopoulos G.K. (2013). Expression of hepatocyte epidermal growth factor receptor, FAS and glypican 3 in EpCAM-positive regenerative clusters of hepatocytes, cholangiocytes, and progenitor cells in human liver failure. Hum. Pathol..

[bib21] He Z., Zhang H., Zhang X., Xie D., Chen Y., Wangensteen K.J., Ekker S.C., Firpo M., Liu C., Xiang D. (2010). Liver xeno-repopulation with human hepatocytes in Fah-/-Rag2-/- mice after pharmacological immunosuppression. Am. J. Pathol..

[bib22] Heron, M. (2012). Deaths: Leading causes for 2009. National Vital Statistics Reports. October 26, 2012. http://www.cdc.gov/nchs/data/nvsr/nvsr61/nvsr61_07.pdf. 24964584

[bib23] Huch M., Bonfanti P., Boj S.F., Sato T., Loomans C.J., van de Wetering M., Sojoodi M., Li V.S., Schuijers J., Gracanin A. (2013). Unlimited in vitro expansion of adult bi-potent pancreas progenitors through the Lgr5/R-spondin axis. EMBO J..

[bib24] Huch M., Dorrell C., Boj S.F., van Es J.H., Li V.S., van de Wetering M., Sato T., Hamer K., Sasaki N., Finegold M.J. (2013). In vitro expansion of single Lgr5+ liver stem cells induced by Wnt-driven regeneration. Nature.

[bib25] Hussein S.M., Batada N.N., Vuoristo S., Ching R.W., Autio R., Närvä E., Ng S., Sourour M., Hämäläinen R., Olsson C. (2011). Copy number variation and selection during reprogramming to pluripotency. Nature.

[bib26] Jorns C., Gramignoli R., Saliem M., Zemack H., Mörk L.M., Isaksson B., Nowak G., Ericzon B.G., Strom S., Ellis E. (2014). Strategies for short-term storage of hepatocytes for repeated clinical infusions. Cell Transplant..

[bib27] Jung P., Sato T., Merlos-Suárez A., Barriga F.M., Iglesias M., Rossell D., Auer H., Gallardo M., Blasco M.A., Sancho E. (2011). Isolation and in vitro expansion of human colonic stem cells. Nat. Med..

[bib28] Kamath B.M., Spinner N.B., Rosenblum N.D. (2013). Renal involvement and the role of Notch signalling in Alagille syndrome. Nat. Rev. Nephrol..

[bib29] Laurent L.C., Ulitsky I., Slavin I., Tran H., Schork A., Morey R., Lynch C., Harness J.V., Lee S., Barrero M.J. (2011). Dynamic changes in the copy number of pluripotency and cell proliferation genes in human ESCs and iPSCs during reprogramming and time in culture. Cell Stem Cell.

[bib30] Lawless M.W., Mankan A.K., Gray S.G., Norris S. (2008). Endoplasmic reticulum stress—a double edged sword for Z alpha-1 antitrypsin deficiency hepatoxicity. Int. J. Biochem. Cell Biol..

[bib31] Liang G., Zhang Y. (2013). Genetic and epigenetic variations in iPSCs: potential causes and implications for application. Cell Stem Cell.

[bib32] Lund R.J., Närvä E., Lahesmaa R. (2012). Genetic and epigenetic stability of human pluripotent stem cells. Nat. Rev. Genet..

[bib33] Martins-Taylor K., Nisler B.S., Taapken S.M., Compton T., Crandall L., Montgomery K.D., Lalande M., Xu R.H. (2011). Recurrent copy number variations in human induced pluripotent stem cells. Nat. Biotechnol..

[bib34] Massagué J., Seoane J., Wotton D. (2005). Smad transcription factors. Genes Dev..

[bib35] Mayshar Y., Ben-David U., Lavon N., Biancotti J.C., Yakir B., Clark A.T., Plath K., Lowry W.E., Benvenisty N. (2010). Identification and classification of chromosomal aberrations in human induced pluripotent stem cells. Cell Stem Cell.

[bib36] McCright B., Lozier J., Gridley T. (2002). A mouse model of Alagille syndrome: Notch2 as a genetic modifier of Jag1 haploinsufficiency. Development.

[bib37] Michalopoulos G.K. (2014). The liver is a peculiar organ when it comes to stem cells. Am. J. Pathol..

[bib38] Mitaka T. (1998). The current status of primary hepatocyte culture. Int. J. Exp. Pathol..

[bib39] Pera M.F. (2011). Stem cells: The dark side of induced pluripotency. Nature.

[bib40] Rashid S.T., Corbineau S., Hannan N., Marciniak S.J., Miranda E., Alexander G., Huang-Doran I., Griffin J., Ahrlund-Richter L., Skepper J. (2010). Modeling inherited metabolic disorders of the liver using human induced pluripotent stem cells. J. Clin. Invest..

[bib41] Sato T., Vries R.G., Snippert H.J., van de Wetering M., Barker N., Stange D.E., van Es J.H., Abo A., Kujala P., Peters P.J., Clevers H. (2009). Single Lgr5 stem cells build crypt-villus structures in vitro without a mesenchymal niche. Nature.

[bib42] Sato T., Stange D.E., Ferrante M., Vries R.G., Van Es J.H., Van den Brink S., Van Houdt W.J., Pronk A., Van Gorp J., Siersema P.D., Clevers H. (2011). Long-term expansion of epithelial organoids from human colon, adenoma, adenocarcinoma, and Barrett’s epithelium. Gastroenterology.

[bib43] Schaub J.R., Malato Y., Gormond C., Willenbring H. (2014). Evidence against a stem cell origin of new hepatocytes in a common mouse model of chronic liver injury. Cell Rep..

[bib44] Schmelzer E., Zhang L., Bruce A., Wauthier E., Ludlow J., Yao H.L., Moss N., Melhem A., McClelland R., Turner W. (2007). Human hepatic stem cells from fetal and postnatal donors. J. Exp. Med..

[bib45] Schwank G., Koo B.K., Sasselli V., Dekkers J.F., Heo I., Demircan T., Sasaki N., Boymans S., Cuppen E., van der Ent C.K. (2013). Functional repair of CFTR by CRISPR/Cas9 in intestinal stem cell organoids of cystic fibrosis patients. Cell Stem Cell.

[bib46] Shan J., Schwartz R.E., Ross N.T., Logan D.J., Thomas D., Duncan S.A., North T.E., Goessling W., Carpenter A.E., Bhatia S.N. (2013). Identification of small molecules for human hepatocyte expansion and iPS differentiation. Nat. Chem. Biol..

[bib47] Shin S., Walton G., Aoki R., Brondell K., Schug J., Fox A., Smirnova O., Dorrell C., Erker L., Chu A.S. (2011). Foxl1-Cre-marked adult hepatic progenitors have clonogenic and bilineage differentiation potential. Genes Dev..

[bib48] Stoller J.K., Aboussouan L.S. (2005). Alpha1-antitrypsin deficiency. Lancet.

[bib49] Sugimoto H., Yang C., LeBleu V.S., Soubasakos M.A., Giraldo M., Zeisberg M., Kalluri R. (2007). BMP-7 functions as a novel hormone to facilitate liver regeneration. FASEB J..

[bib50] Vilarinho S., Lifton R.P. (2012). Liver transplantation: from inception to clinical practice. Cell.

[bib51] Wandel C., Böcker R., Böhrer H., Browne A., Rügheimer E., Martin E. (1994). Midazolam is metabolized by at least three different cytochrome P450 enzymes. Br. J. Anaesth..

[bib52] Wiegerinck C.L., Janecke A.R., Schneeberger K., Vogel G.F., van Haaften-Visser D.Y., Escher J.C., Adam R., Thöni C.E., Pfaller K., Jordan A.J. (2014). Loss of syntaxin 3 causes variant microvillus inclusion disease. Gastroenterology.

[bib53] Wu A.L., Coulter S., Liddle C., Wong A., Eastham-Anderson J., French D.M., Peterson A.S., Sonoda J. (2011). FGF19 regulates cell proliferation, glucose and bile acid metabolism via FGFR4-dependent and independent pathways. PLoS ONE.

[bib54] Xu J., Lamouille S., Derynck R. (2009). TGF-beta-induced epithelial to mesenchymal transition. Cell Res..

[bib55] Yanger K., Zong Y., Maggs L.R., Shapira S.N., Maddipati R., Aiello N.M., Thung S.N., Wells R.G., Greenbaum L.E., Stanger B.Z. (2013). Robust cellular reprogramming occurs spontaneously during liver regeneration. Genes Dev..

[bib56] Yanger K., Knigin D., Zong Y., Maggs L., Gu G., Akiyama H., Pikarsky E., Stanger B.Z. (2014). Adult hepatocytes are generated by self-duplication rather than stem cell differentiation. Cell Stem Cell.

[bib57] Yoon S.M., Gerasimidou D., Kuwahara R., Hytiroglou P., Yoo J.E., Park Y.N., Theise N.D. (2011). Epithelial cell adhesion molecule (EpCAM) marks hepatocytes newly derived from stem/progenitor cells in humans. Hepatology.

